# Oncogenic KRAS represses the dependence receptor UNC5C *via* ERK2-FOS signaling in non-small cell lung cancer

**DOI:** 10.1016/j.jbc.2026.113262

**Published:** 2026-06-19

**Authors:** Deyu Wang, Man Xiao, Kaicheng Zhou, Peng Yang, Xiting Huang, Runze Miao, Jiajun Fan, Xuyao Zhang, Shaofei Wang

**Affiliations:** 1 Department of Cellular and Genetic Medicine, School of Basic Medical Sciences, Fudan University, Shanghai, P.R. China; 2 Molecular Diagnostic Laboratory, Department of Clinical Laboratory, Shanghai Children’s Hospital, School of Medicine, Shanghai Jiao Tong University, Shanghai, P.R. China; 3 Department of Biological Medicines, School of Pharmaceutical Sciences, Shanghai Engineering Research Center of Immunotherapeutics, Fudan University, Shanghai, P.R. China

**Keywords:** dependence receptors, KRAS, NSCLC, tumor suppressor genes, UNC5C

## Abstract

KRAS mutation is one of the most prevalent oncogenic driver mutations in NSCLC. UNC5C, as a member of dependence receptors of netrin-1, is a conditional tumor suppressor in cancer progression and metastasis *via* inducing apoptosis. Despite UNC5C has been intensively reported to be downregulated in multiple types of malignancies, the mechanism of UNC5C loss or downregulation in cancer remains unclear. In this study, we identified UNC5C as a downstream effector of oncogenic KRAS signaling pathway. We found that oncogenic KRAS suppressed UNC5C and inhibition of oncogenic KRAS upregulated UNC5C in KRAS-mutant NSCLC. Mechanically, oncogenic KRAS-mediated downregulation of UNC5C was dependent on the activation of the RAF/MEK/ERK cascade rather than the PI3K/AKT/mTOR pathway. More specifically, ERK2, but not ERK1, was involved in the control of UNC5C expression. Critically, FOS, a downstream transcription factor of the ERK pathway, was responsible for the transcriptional repression of UNC5C in KRAS-mutant NSCLC. In addition, UNC5C, rather than other dependence receptors of netrin-1, was most strongly downregulated in NSCLC and functioned as a tumor suppressor. In conclusion, we reported oncogenic KRAS-mediated transcriptional suppression of UNC5C and deciphered the exact underlying molecular mechanism in NSCLC, thus providing novel insights into the interplay between oncogenes and tumor suppressor genes in KRAS-driven NSCLC.

Gain-of-function mutation or overexpression of oncogenes (OCGs) and loss-of-function mutation or downregulation of tumor suppressor genes (TSGs) are dominant drivers of tumorigenesis (1, 2). Therefore, targeting OCGs or TSGs possesses tremendous therapeutic potentials for the treatment of cancer (3, 4). KRAS is a proto-oncogene encoding a small GTPase, which acts as a molecular switch transmitting extracellular signals to multiple downstream effectors to drive cell proliferation, differentiation and survival. Mutations of KRAS, typically occurring in codons G12, G13 or Q61, disrupting GTP hydrolysis (5) and thus leading to constitutive activation of downstream RAF/MEK/ERK, PI3K/AKT/mTOR and other signaling pathways (6), are genetic drivers of multiple types of human cancers, in particular pancreatic ductal adenocarcinoma, colorectal cancer, and non-small cell lung cancer (NSCLC) (7, 8). Recently, KRAS^G12C^-specific inhibitors, including sotorasib (AMG-510) (9, 10) and adagrasib (MRTX849) (11), which selectively trap the oncoprotein in an inactive GDP-bound conformation, have been clinically approved for the treatment of adult patients with KRAS^G12C^-mutated locally advanced or metastatic NSCLC, thereby opening a new era of the clinical management of KRAS-driven lung cancer (12, 13, 14). However, the overall response rate to both KRAS inhibitors in patients with KRAS^G12C^-mutant NSCLC is limited, ranging from 37.1% to 42.9% (15, 16), thus making it necessary to further characterize the molecular response of NSCLC cells to KRAS inhibitor treatment. In addition, identifying crucial downstream effector molecules of oncogenic KRAS may provide novel insights for target discovery for the treatment of KRAS-driven NSCLC.

The uncoordinated-5 (UNC5) homologue family, including UNC5A, UNC5B, UNC5C and UNC5D, and deleted in colorectal cancer (DCC), are functional dependence receptors (DRs) depending on the availability of their ligand netrin-1, which intrinsically trigger apoptosis in the absence of ligand binding and actively promote cell survival in the presence of netrin-1 (17, 18, 19, 20). In light of their aberrant downregulation in the vast majority of human cancers, DRs function as conditional tumor suppressors in cancer progression and metastasis *via* eliciting apoptosis in the context of a lack of their corresponding ligands. In contrast, netrin-1, which is frequently upregulated in cancer, generally functions as an oncogene in cancer (18, 21, 22, 23, 24). Moreover, in recent years, netrin-1 and its DRs have attracted increasing attention in the field of cancer therapy (19, 20, 23, 25, 26) and targeting the interaction between netrin-1 and its DRs has emerged as a potential therapeutic strategy for the treatment of cancer (27, 28, 29, 30, 31, 32, 33, 34, 35, 36). More specifically, due to their apoptosis-inducing function in the absence of ligand binding, UNC5 homologue family receptors and DCC may serve as alternative targets for the development of cancer therapies, thus making it meaningful to decipher the molecular regulatory mechanisms of these DRs. Among them, UNC5C is frequently downregulated in multiple types of cancer, especially colon adenocarcinoma (COAD), rectum adenocarcinoma (READ), prostate adenocarcinoma (PRAD), lung adenocarcinoma (LUAD) and lung squamous cell carcinoma (LUSC) (37). The loss or downregulation of UNC5C expression is mainly attributed to the methylation of its promoter in human colorectal cancer (38, 39, 40, 41, 42) and renal cell carcinoma (43). The reduction of UNC5C expression in colorectal cancer (44) and renal cell carcinoma (43) is also partially associated with loss of heterozygosity. Genomic fusion of UNC5C and SNX9 leads to downregulation of UNC5C expression in prostate cancer (45). In addition, RHOX5 homeodomain protein mediates transcriptional repression of UNC5C expression (46). However, although it is generally recognized that the expression of UNC5C in cancer is regulated at genomic, epigenetic and transcriptional levels (37), the underlying mechanism mediating the loss or downregulation of UNC5C expression in cancer remains largely elusive.

Our preliminary study suggested that oncogenic KRAS induced transcriptional repression of UNC5C in NSCLC. However, the underlying molecular mechanism by which oncogenic KRAS regulates UNC5C expression remains unknown. Previous findings have indicated that RAS-directed cell transformation by driving silencing of TSGs through inducing genomic methylation (47, 48, 49, 50, 51), thus prompting us to examine the methylation status of the promoter region of UNC5C in NSCLC. However, distinct from COAD and READ, UNC5C was not hypermethylated and its expression was not closely correlated with methylation of its promoter in LUAD and LUSC (37), implying the involvement of previously unspecified mechanisms in mediating the downregulation of UNC5C expression in NSCLC. To be specific, the regulation of UNC5C expression is complicated, probably partially depending on the status of underlying signaling transduction pathways and downstream gene transcriptional regulatory networks. Herein, in the current study, we found that inhibition of oncogenic KRAS significantly upregulated UNC5C expression in KRAS-mutant NSCLC. Mechanically, oncogenic KRAS-mediated transcriptional suppression of UNC5C was dependent on activation of the RAF/MEK/ERK cascade rather than the PI3K/AKT/mTOR pathway. More specifically, ERK2, but not ERK1, was involved in the regulation of UNC5C expression. Critically, activator protein-1 (AP-1) transcription factor FOS, as a downstream transcription factor of the ERK pathway, was most likely responsible for the transcriptional repression of UNC5C in KRAS-mutant NSCLC. Therefore, oncogenic KRAS transcriptionally downregulated the expression of UNC5C in KRAS-driven NSCLC by activating the RAF/MEK/ERK signaling pathway, in particular its downstream AP-1 transcription factor FOS. In conclusion, this study elucidated a previously unspecified regulatory mechanism of conditional tumor suppressor UNC5C by oncogenic KRAS signaling and thus provided innovative insights into the interplay between OCGs and TSGs in cancer.

## Results

### KRAS inhibitor induced upregulation of UNC5C in KRAS-mutant NSCLC

Data mining of previous RNA-seq data (52) indicated that *UNC5C* was the most significantly and robustly upregulated gene in KRAS-mutant NCI-H2009 cells after KRAS knockdown (Fig. S1). To be specific, a total of 260 common differentially expressed genes (DEGs), including 148 upregulated genes and 112 downregulated genes, were identified after KRAS knockdown in KRAS-mutant NCI-H2009 cells (Fig. S1*A*, middle panel). Notably, UNC5C, was the most strongly and exclusively upregulated gene after KRAS knockdown, while KRAS was among the most significantly downregulated genes (Fig. S1*A*, left panel and right panel; Fig. S1, *B* and *C*). To determine the downstream signaling pathways enriched after oncogenic KRAS knockdown, we performed the hallmark GSEA on RNA-seq data of KRAS^G12A^-mutant NCI-H2009 cells after KRAS knockdown. Interestingly, the KRAS signaling pathway was negatively enriched after oncogenic KRAS knockdown, whereas the PI3K/AKT/mTOR signaling pathway was not significantly changed (Fig. S1*D*). More specifically, KRAS signaling UP was among the most negatively enriched pathways in response to KRAS knockdown with a normalized enrichment score (NES) of −1.498 and −1.535, while KRAS signaling DOWN pathway was among the most positively enriched pathways with a normalized enrichment score (NES) of 1.503 and 1.416 (Fig. S1*E*). These data indicated that KRAS signaling was significantly downregulated and UNC5C was strongly upregulated after oncogenic KRAS knockdown, suggesting a possible negative correlation between UNC5C expression and KRAS signaling. We thus proposed that UNC5C was a potential downstream effector of oncogenic KRAS. In parallel, UNC5C is a dependence receptor capable of inducing apoptosis in the absence of ligand binding, making it reasonable to speculate UNC5C as a potential downstream effector of KRAS inhibition-mediated tumor suppression.

**Figure 1:**
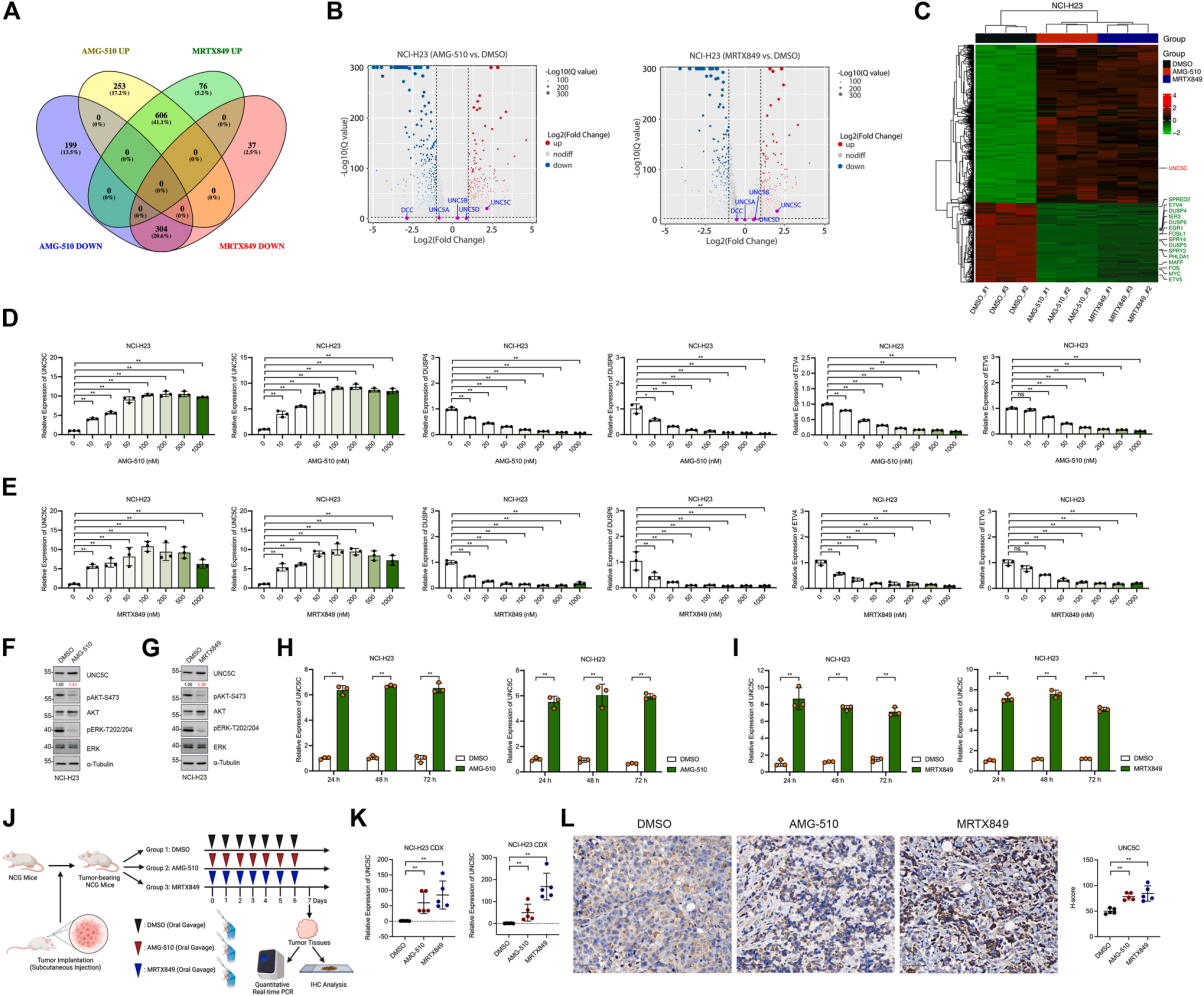
**KRAS inhibitor induced upregulation of UNC5C in KRAS-mutant NSCLC.***A*, Venn diagram illustrating the commonly upregulated and downregulated genes induced by treatment with KRAS^G12C^-specific inhibitor AMG-510 (1 μM) or MRTX849 (100 nM) for 24 h in KRAS^G12C^-mutant NCI-H23 cells, as indicated by RNA-seq. *B*, Volcano plots showing the DEGs, in particular *UNC5C*, in KRAS^G12C^-mutant NCI-H23 cells after exposure to KRAS^G12C^-specific inhibitor AMG-510 (*left*) or MRTX849 (*right*) for 24 h, as indicated by RNA-seq. *C*, heatmap illustrating the expression of DEGs, in particular *UNC5C* and ERK-dependent genes, in KRAS^G12C^-mutant NCI-H23 cells after exposure to KRAS^G12C^-specific inhibitor AMG-510 or MRTX849 for 24 h, as indicated by RNA-seq. *D*, the relative expression of *UNC5*C and ERK-dependent genes (*DUSP4*, *DUSP6*, *ETV4*, and *ETV5*) after KRAS^G12C^-mutant NCI-H23 cells were treated by various concentrations (0, 10, 20, 50, 100, 200, 500, 1000 nM) of KRAS^G12C^-specific inhibitor AMG-510 as indicated, as assessed by quantitative real-time PCR. *E*, the relative expression of *UNC5C* and ERK-dependent genes (*DUSP4*, *DUSP6*, *ETV4*, and *ETV5*) after KRAS^G12C^-mutant NCI-H23 cells were treated by various concentrations (0, 10, 20, 50, 100, 200, 500, 1000 nM) of KRAS^G12C^-specific inhibitor MRTX849 as indicated, as assessed by quantitative real-time PCR. *F*, the expression of UNC5C protein in KRAS^G12C^-mutant NCI-H23 cells after treated by KRAS^G12C^-specific inhibitor AMG-510 (1 μM) for 24 h, as indicated by immunoblot analysis. *G*, the expression of UNC5C protein in KRAS^G12C^-mutant NCI-H23 cells after treated by KRAS^G12C^-specific inhibitor MRTX849 (100 nM) for 24 h, as indicated by immunoblot analysis. *H*, the relative expression of *UNC5C* in KRAS^G12C^-mutant NCI-H23 cells after treated by KRAS^G12C^-specific inhibitor AMG-510 (1 μM) for 24 h, 48 h and 72 h, as assessed by quantitative real-time PCR. *I*, the relative expression of *UNC5C* in KRAS^G12C^-mutant NCI-H23 cells after treated by KRAS^G12C^-specific inhibitor MRTX849 (100 nM) for 24 h, 48 h and 72 h, as assessed by quantitative real-time PCR. *J*, schematic diagram showing the establishment of NCI-H23 xenograft models using immunodeficient NCG mice and the assessment of UNC5C expression after treatment with either AMG-510 or MRTX849 (10 mg/kg body weight) for seven consecutive days (N = 5 mice/group). *K*, the relative expression of *UNC5C* in tumor tissues of NCI-H23 xenografts after tumor-bearing NCG mice were treated by KRAS^G12C^-specific inhibitor AMG-510 or MRTX849 for seven consecutive days, as indicated by quantitative real-time PCR (N = 5 mice/group). *L*, representative photographs of UNC5C IHC staining (*left*) and H-score quantification of UNC5C expression (*right*) in NCI-H23 xenografts tumors of mice after treatment by KRAS^G12C^-specific inhibitor AMG-510 or MRTX849 for seven consecutive days (N = 5 mice/group). Scale bar: 50 μm. ∗*p* < 0.05; ∗∗*p* < 0.01. See also Figures S1 and S2.

To determine KRAS-regulated gene transcriptome profiles in KRAS-driven NSCLC, high-throughput RNA-seq was performed to determine the DEGs in KRAS^G12C^-mutant NCI-H23 cells after clinically available KRAS^G12C^-specific inhibitor treatment (Fig. 1*A*). Herein, a total of 1362 DEGs, including 859 upregulated genes and 503 downregulated genes, were identified after AMG-510 treatment, while a total of 1023 DEGs, including 682 upregulated genes and 341 downregulated genes, were identified after MRTX849 treatment. The commonly shared DEGs induced by AMG-510 and MRTX849 were 606 upregulated genes and 304 downregulated genes. Among the gene encoding members of the UNC5 homologue family DRs, UNC5C was significantly upregulated after KRAS inhibitor exposure, as accompanied by the downregulation of ERK-dependent genes (Fig. 1, *B* and *C*). In contrast, other DRs of netrin-1, including UNC5A, UNC5B, UNC5D, and DCC, were not consistently altered in response to KRAS inhibitors (Fig. 1*B*). To further verify the upregulation of UNC5C induced by the KRAS inhibitor, we also examined the expression of UNC5C at mRNA and protein levels by quantitative real-time PCR and immunoblot analysis, respectively. As expected, the selective KRAS^G12C^ inhibitor, either AMG-510 or MRTX849, upregulated UNC5C at the transcriptional level in a dose-dependent manner in KRAS^G12C^-mutant NSCLC (Fig. 1, *D* and *E*). In parallel, ARS-1620, the direct precursor to the clinically approved KRAS^G12C^ inhibitor, also dose-dependently induced UNC5C overexpression at the transcriptional level in KRAS^G12C^-mutant NSCLC (Fig. S2*A*). Similarly, BI-2865, a pan-KRAS inhibitor, dose-dependently increased *UNC5C* gene transcription in KRAS-mutant NSCLC (Fig. S2, *B* and *C*). Moreover, KRAS inhibitor AMG-510 or MRTX849 induced the upregulation of UNC5C expression at the protein level (Fig. 1, *F* and *G*). Strikingly, KRAS inhibitor treatment elevated the mRNA levels of UNC5C not only at an earlier time point but also at a later time point, indicating that KRAS inhibitor mediated sustainable upregulation of UNC5C (Fig. 1, *H* and *I*). To determine whether the KRAS inhibitor could upregulate UNC5C expression *in vivo*, we examined the expression of UNC5C in tumor tissues of murine models of KRAS-mutant NSCLC after KRAS inhibitor treatment (Fig. 1*J*). Consistent with *in vitro* results, KRAS inhibitor AMG-510 or MRTX849 mediated the upregulation of UNC5C in tumor tissues at the transcriptional level *in vivo* (Fig. 1*K*). Immunohistochemistry (IHC) analysis also indicated that the KRAS inhibitor significantly elevated tumoral UNC5C expression (Fig. 1*L*). Taken together, these data demonstrated that the KRAS inhibitor induced the upregulation of UNC5C expression both *in vitro* and *in vivo* in KRAS-mutant NSCLC.

### Oncogenic KRAS signaling mediated the transcriptional suppression of UNC5C expression

**Figure 2:**
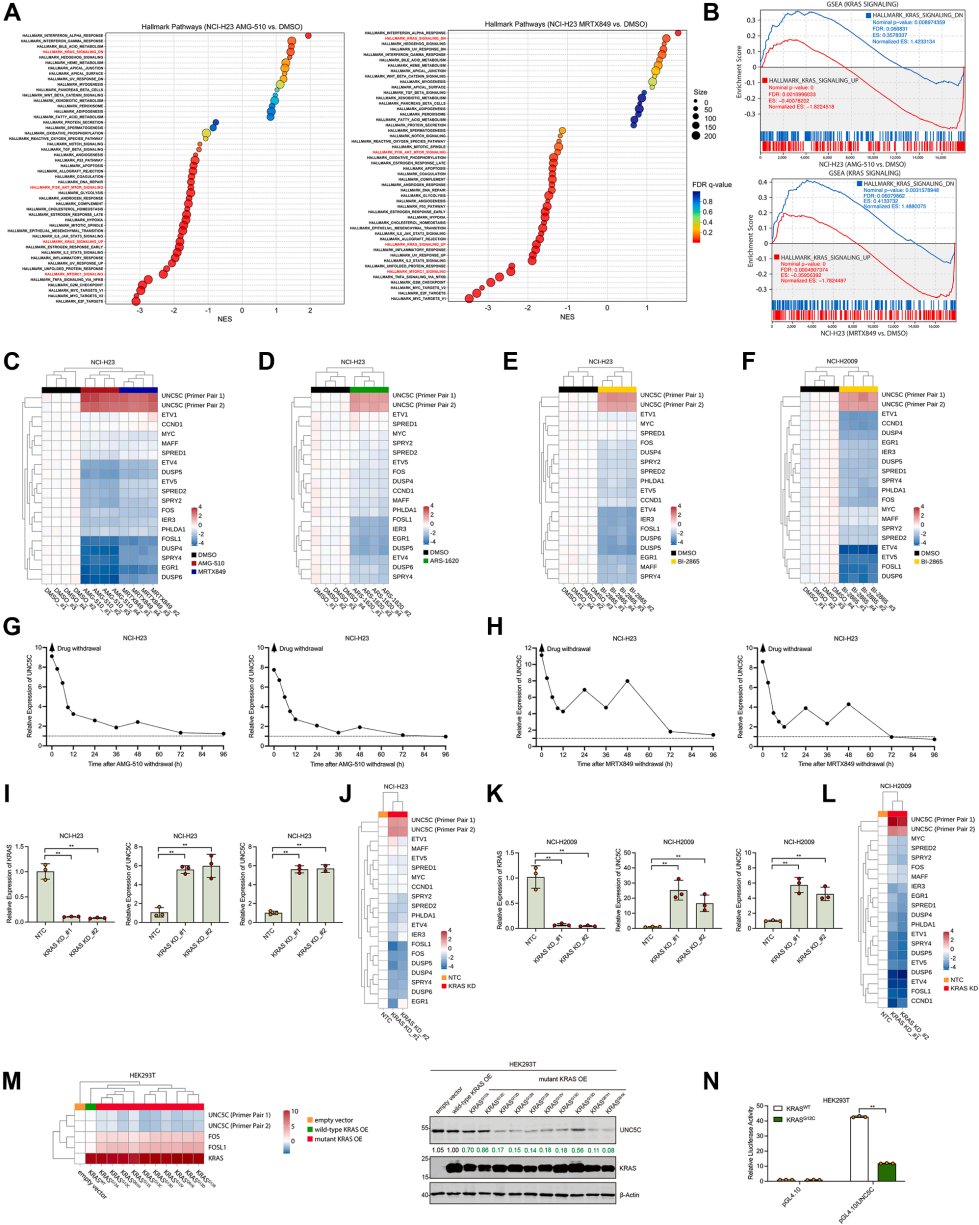
**Oncogenic KRAS signaling mediated the transcriptionalsuppressionof UNC5C expression.***A*, hallmark pathways from gene set enrichment analysis (GSEA) of genes affected by AMG-510 (*left*) or MRTX849 (*right*) treatment in NCI-H23 cells, as indicated by RNA-seq. *B*, GSEA analysis of KRAS Signaling Up and KRAS Signaling DOWN signatures in NCI-H23 cells treated with AMG-510 (*upper panel*) or MRTX849 (*lower panel*), as indicated by RNA-seq. Normalized enrichment score (NES) for each pathway were indicated in corresponding color. *C*, heatmap illustrating the relative expression of *UNC5C* and ERK-dependent genes in NCI-H23 cells after treatment by AMG-510 (1 μM) or MRTX849 (100 nM) for 24 h, as indicated by quantitative real-time PCR. N = 4 independent biological replicates. *D*, heatmap illustrating the relative expression of *UNC5C* and ERK-dependent genes in NCI-H23 cells after treatment by ARS-1620 (1 μM) for 24 h, as indicated by quantitative real-time PCR. N = 4 independent biological replicates. *E*, heatmap illustrating the relative expression of *UNC5C* and ERK-dependent genes in KRAS^G12C^-mutant NCI-H23 cells after treatment by pan-KRAS inhibitor BI-2865 (1 μM) for 24 h, as indicated by quantitative real-time PCR. N = 4 independent biological replicates. *F*, heatmap illustrating the relative expression of *UNC5C* and ERK-dependent genes in KRAS^G12A^-mutant NCI-H2009 cells after treatment by pan-KRAS inhibitor BI-2865 (1 μM) for 24 h, as indicated by quantitative real-time PCR. N = 4 independent biological replicates. *G*, the relative expression of *UNC5C* at mRNA levels in NCI-H23 cells after withdrawal of AMG-510 (1 μM) treatment at indicated time points, as indicated by quantitative real-time PCR. *H*, the relative expression of *UNC5C* at mRNA levels in NCI-H23 cells after withdrawal of MRTX849 (100 nM) treatment at indicated time points, as indicated by quantitative real-time PCR. *I*, bar graph showing the relative expression of *KRAS* and *UNC5C* at mRNA levels after KRAS knockdown in NCI-H23 cells. *J*, heatmap depicting the relative expression of *UNC5C* and ERK-dependent genes at mRNA levels in response to KRAS knockdown in NCI-H23 cells. *K*, bar graph showing the relative expression of *KRAS* and *UNC5C* at mRNA levels after KRAS knockdown in NCI-H2009 cells. *L*, heatmap depicting the relative expression of *UNC5C* and ERK-dependent genes at mRNA levels in response to KRAS knockdown in NCI-H2009 cells. *M*, the relative expression of UNC5C at mRNA (*left*) and protein (*right*) levels in HEK293T cells overexpressing wild-type or mutant KRAS. *N*, the relative luciferase activity driven the empty vector or wild-type *UNC5C* promoter in the presence of either KRAS^WT^ or KRAS^G12C^ co-transfection. HEK293T cells were co-transfected with the pGL4.10-*UNC5C* promoter luciferase reporter or empty vector and KRAS^WT^ or KRAS^G12C^ plasmid for 48 h. ∗∗*p* < 0.01. See also Figures S3–S5. KD, knockdown; OE, overexpression.

To determine the downstream pathways enriched after KRAS inhibitor treatment, we performed the hallmark GSEA on RNA-seq data of NCI-H23 cells upon AMG-510 or MRTX849 exposure and found that KRAS and mTORC1 signaling pathways were negatively enriched in response to KRAS inhibitor treatment (Fig. 2*A*). Notably, KRAS signaling UP and KRAS signaling DOWN pathways were among the most negatively and positively enriched pathways in response to AMG-510 treatment, with a normalized enrichment score (NES) of −1.822 and 1.423, respectively (Fig. 2*B*, upper panel). Similarly, these two pathways were also among the most negatively and positively enriched pathways after MRTX849 treatment, with a NES of −1.782 and 1.488, respectively (Fig. 2*B*, lower panel). These data indicated that KRAS signaling was significantly downregulated upon AMG-510 or MRTX849 treatment. Further studies indicated that the clinically approved KRAS^G12C^-specific inhibitors, either AMG-510 or MRTX849, induced upregulation of *UNC5C* and downregulation of ERK-dependent genes (Fig. 2*C*). In parallel, the KRAS^G12C^ inhibitor ARS-1620 (Fig. 2*D*) and the pan-KRAS inhibitor BI-2865 (Fig. 2, *E* and *F*) also resulted in an increase of *UNC5C* expression, as accompanied by decreases of ERK-dependent genes. These results indicated that UNC5C upregulation was a recurrent transcriptional event in response to KRAS inhibitor treatment. However, in KRAS-mutant cell lines exhibiting complete loss of UNC5C expression at mRNA levels, KRAS inhibitor treatment failed to induce re-expression of UNC5C (Fig. S3), suggesting the existence of other unspecified mechanisms mediating the complete loss of UNC5C expression in some of KRAS-mutant cancers. Next, we examined whether the upregulation of UNC5C expression was a specific molecular response to KRAS inhibitor by drug withdrawal experiment (Fig. 2, *G* and *H*). Following the removal of KRAS inhibitor treatment, the expression of *UNC5C* was consequently downregulated and restored back to basal levels within 4 days. These findings indicated the specificity and reversibility of KRAS inhibitor-mediated upregulation of *UNC5C*.

To further explore whether KRAS inhibitor-mediated upregulation of UNC5C was dependent on suppression of oncogenic KRAS signaling pathway, we determined the expression of UNC5C after knockdown of KRAS by two individual siRNAs in KRAS-mutant NSCLC cells and found that KRAS knockdown substantially elevated UNC5C expression at the transcriptional level in NCI-H23 (Fig. 2, *I* and *J*) and NCI-H2009 cells (Fig. 2, *K* and *L*). Moreover, siRNA specifically targeting KRAS^G12C^ mutation, rather than siRNA targeting wild-type KRAS, significantly induced the overexpression of UNC5C in KRAS^G12C^-mutant NCI-H23 cells compared with non-targeting control (Fig. S4). We also determined whether oncogenic KRAS could affect UNC5C expression at mRNA and protein levels. As expected, overexpression of mutant KRAS but not wild-type KRAS significantly decreased the expression of *UNC5C* at both the mRNA and protein levels (Fig. 2*M*), implying that oncogenic KRAS directed the transcriptional repression of UNC5C expression. In addition, to further confirm the transcriptional regulation of UNC5C by oncogenic KRAS, we performed a dual luciferase reporter assay after co-transfection of oncogenic KRAS^G12C^ or wild-type KRAS and a luciferase reporter construct harboring *UNC5C* promoter fragment. Interestingly, co-transfection of KRAS^G12C^ substantially decreased the luciferase activity driven by the promoter of *UNC5C*, suggesting that oncogenic KRAS suppressed the transactivation of *UNC5C* (Fig. 2*N*). Taken together, these data suggested that oncogenic KRAS signaling directed the transcriptional suppression of UNC5C expression in KRAS-mutant NSCLC.

Given that both transcriptional regulation and post-translational control of UNC5C jointly determine the protein level of UNC5C, protein stability analysis of UNC5C allows for a clearer dissection of post-translational regulation of UNC5C upon oncogenic KRAS overexpression or KRAS inhibitor treatment and is crucial for establishing a more comprehensive view of the regulatory relationship between oncogenic KRAS signaling and UNC5C protein dynamics. Therefore, we determined the protein stability of UNC5C upon overexpression of mutant KRAS (Fig. S5*A*) or KRAS inhibitor treatment (Fig. S5*B*). Interestingly, compared with overexpression of wild-type KRAS, overexpression of oncogenic KRAS^G12C^ significantly downregulated the protein level of UNC5C but prolonged the protein half-life of UNC5C in HEK293T cells (Fig. S5*A*). In contrast, inhibition of oncogenic KRAS by KRAS inhibitor significantly upregulated the protein level of UNC5C but accelerated the turnover of UNC5C protein in KRAS-mutant NCI-H23 cells (Fig. S5*B*). These data indicated that oncogenic KRAS regulated UNC5C expression not only through regulation of gene transcription but also *via* control of protein stability. However, between these two distinct regulatory mechanisms, transcriptional control served as the major driving force for modulation of UNC5C protein expression by oncogenic KRAS, while protein stability modulation by oncogenic KRAS—either specific or non-specific—might probably constitute an intricate negative feedback loop to stabilize the expression of UNC5C protein.

### The RAF/MEK/ERK pathway but not the PI3K/AKT/mTOR pathway was involved in oncogenic KRAS-mediated suppression of UNC5C

**Figure 3:**
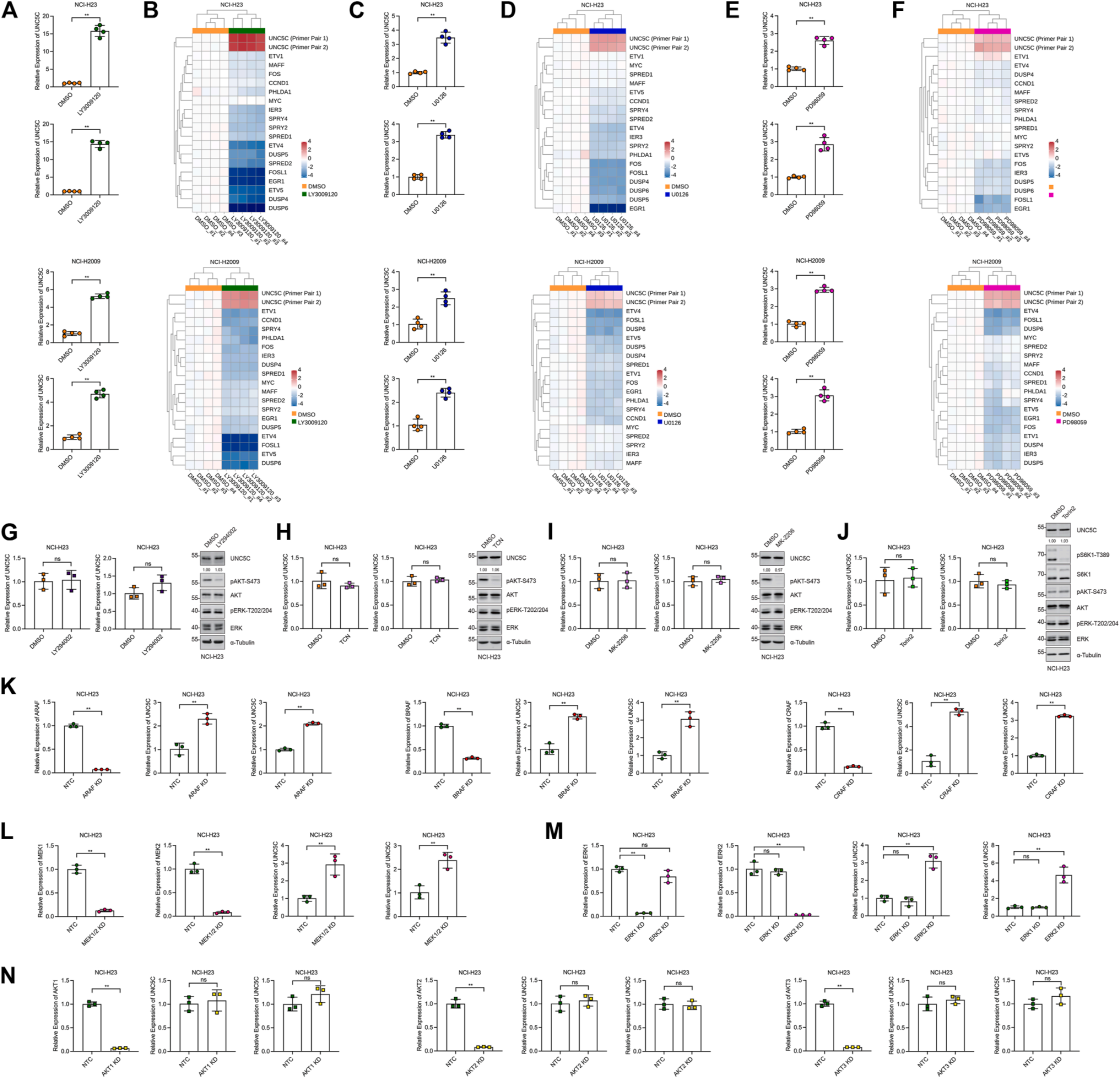
**TheRAF/MEK/ERK pathway but notthePI3K/AKT/mTOR pathway was involved in the control of UNC5C expression.***A*, bar graph showing the relative expression of *UNC5C* in NCI-H23 (*upper panel*) and NCI-H2009 (*lower panel*) cells after exposure to pan-RAF inhibitor LY3009120 (10 μM) for 24 h, as indicated by quantitative real-time PCR. *B*, heatmap illustrating the relative expression of *UNC5C* and ERK-dependent genes in NCI-H23 (*upper panel*) and NCI-H2009 (*lower panel*) cells after exposure to pan-RAF inhibitor LY3009120 (10 μM) for 24 h, as indicated by quantitative real-time PCR. N = 4 independent biological replicates. *C*, bar graph showing the relative expression of *UNC5C* in NCI-H23 (*upper panel*) and NCI-H2009 (*lower panel*) cells after exposure to MEK/ERK inhibitor U0126 (10 μM) for 24 h, as indicated by quantitative real-time PCR. *D*, heatmap illustrating the relative expression of *UNC5C* and ERK-dependent genes in NCI-H23 cells (*upper panel*) and NCI-H2009 (*lower panel*) after exposure to MEK/ERK inhibitor U0126 (10 μM) for 24 h, as indicated by quantitative real-time PCR. N = 4 independent biological replicates. *E*, bar graph showing the relative expression of *UNC5C* in in NCI-H23 (*upper panel*) and NCI-H2009 (*lower panel*) cells after exposure to MEK/ERK inhibitor PD98059 (20 μM) for 24 h, as indicated by quantitative real-time PCR. *F*, heatmap illustrating the relative expression of *UNC5C* and ERK-dependent genes in NCI-H23 (*upper panel*) and NCI-H2009 (*lower panel*) cells after exposure to MEK/ERK inhibitor PD98059 (20 μM) for 24 h, as indicated by quantitative real-time PCR. N = 4 independent biological replicates. *G*, the relative expression of UNC5C at mRNA (*left*) and protein (*right*) levels in NCI-H23 cells after exposure to PI3K inhibitor LY294002 (10 μM) for 24 h, as indicated by quantitative real-time PCR and immunoblot analysis. *H*, the relative expression of UNC5C at mRNA (*left*) and protein (*right*) levels in NCI-H23 cells after exposure to AKT inhibitor TCN (1 μM) for 24 h, as indicated by quantitative real-time PCR and immunoblot analysis. *I*, the relative expression of UNC5C at mRNA (*left*) and protein (*right*) levels in NCI-H23 cells after exposure to AKT inhibitor MK-2206 (2 μM) for 24 h, as indicated by quantitative real-time PCR and immunoblot analysis. *J*, the relative expression of UNC5C at mRNA (*left*) and protein (*right*) levels in NCI-H23 cells after exposure to mTOR inhibitor Torin2 (100 nM) for 24 h, as indicated by quantitative real-time PCR and immunoblot analysis. *K*, bar graph showing the relative expression of *UNC5C* after knockdown of ARAF (*left*), BRAF (*middle*) or CRAF (*right*) in NCI-H23 cells, as indicated by quantitative real-time PCR. *L*, bar graph showing the relative expression of *UNC5C* after knockdown of MEK1/2 in NCI-H23 cells, as indicated by quantitative real-time PCR. *M*, bar graph showing the relative expression of *UNC5C* after isoform specific knockdown of ERK in NCI-H23 cells, as indicated by quantitative real-time PCR. *N*, bar graph showing the relative expression of *UNC5C* after knockdown of AKT1 (*left*), AKT2 (*middle*) or AKT3 (*right*) in NCI-H23 cells, as indicated by quantitative real-time PCR. ∗∗*p* < 0.01. See also Figure S6. TCN, Triciribine.

Next, we explored the downstream signaling pathway of oncogenic KRAS responsible for the control of UNC5C expression in NSCLC. To test whether UNC5C expression was dependent on the PI3K/AKT/mTOR or the RAF/MEK/ERK pathway, we examined the expression of UNC5C in KRAS-mutant NSCLC cells after exposure to corresponding pharmacological inhibitors. Notably, LY3009120 (Fig. 3, *A* and *B*), a pan-RAF inhibitor, as well as MEK/ERK inhibitors, including U0126 (Fig. 3, *C* and *D*) and PD98059 (Fig. 3, *E* and *F*), significantly upregulated *UNC5C* and downregulated ERK-dependent genes at mRNA levels in KRAS-mutant NCI-H23 and NCI-H2009 cells. Also, these inhibitors targeting RAF/MEK/ERK pathway induced sustainable *UNC5C* upregulation in KRAS-driven NSCLC cells (Fig. S6). In contrast, PI3K inhibitor LY294002 (Fig. 3*G*), AKT inhibitor Triciribine (TCN) or MK-2206 (Fig. 3, *H* and *I*), or mTOR inhibitor Torin2 (Fig. 3*J*), could not induce the upregulation of UNC5C expression at the transcriptional or protein level. These data indicated that inhibition of the RAF/MEK/ERK pathway aberrantly upregulated UNC5C expression in KRAS-mutant NSCLC, whereas inhibition of the PI3K/AKT/mTOR cascade failed to augment UNC5C expression. Moreover, knockdown of ARAF, BRAF or CRAF significantly elevated the expression of UNC5C (Fig. 3*K*). In addition, siRNA-mediated knockdown of MEK1/2 aberrantly upregulated UNC5C expression, which further confirmed that the expression of UNC5C was MEK-dependent in KRAS-mutant NSCLC (Fig. 3*L*). To determine the isoform-specific role of ERKs in the modulation of UNC5C expression, we also determined the expression of UNC5C after knockdown of ERK1 and ERK2, respectively (Fig. 3*M*). Interestingly, specific silencing of endogenous ERK2 significantly elevated the expression of UNC5C, whereas depletion of ERK1 failed to upregulate the expression of UNC5C, implying that ERK2 rather than ERK1 was responsible for the transcriptional regulation of UNC5C in KRAS-mutant NSCLC. In contrast, knockdown of AKT1, AKT2 or AKT3 could not substantially upregulate the expression of UNC5C (Fig. 3*N*). Our data suggested that the activation of the RAF/MEK/ERK pathway, but not the PI3K/AKT/mTOR cascade, mediated the downregulation of UNC5C expression in KRAS-mutant NSCLC. Taken together, these findings strongly indicated that UNC5C expression was downregulated through oncogenic KRAS-directed activation of the RAF/MEK/ERK pathway.

### FOS was involved in the transcriptional repression of UNC5C in KRAS-mutant NSCLC

**Figure 4:**
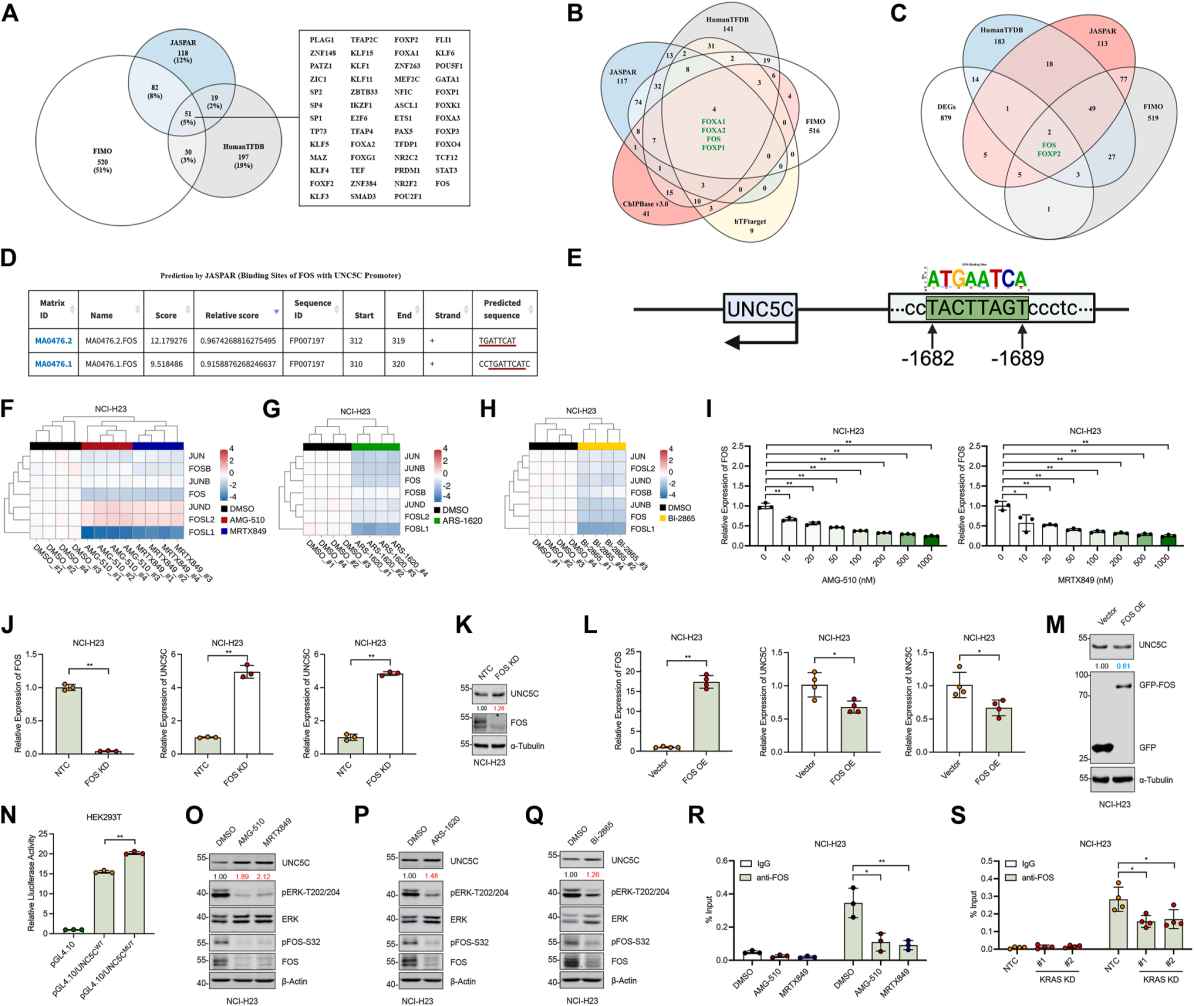
**FOS was involved in the transcriptional repression of UNC5C in KRAS-mutant NSCLC.***A*, Venn Diagram of transcription factors harboring putative binding site within the UNC5C promoter predicted by FIMO, JASPAR, HumanTFDB databases. *B*, Venn Diagram of transcription factors of UNC5C predicted by FIMO, JASPAR, HumanTFDB, ChIPBase v3.0 and hTFtarget. *C*, Venn Diagram of transcription factors of UNC5C predicted by FIMO, JASPAR, HumanTFDB and common DEGs induced by KRAS inhibitor treatment. *D*, putative binding site of AP-1 transcription factor FOS within human UNC5C promoter predicted by JASPAR. *E*, conservative analysis of transcription factor binding site of FOS within the promoter of UNC5C among primates. *F*, Heatmap showing the relative expression of AP-1 family transcription factors in NCI-H23 cells after treatment by AMG-510 (1 μM) or MRTX849 (100 nM) for 24 h, as indicated by quantitative real-time PCR. *G*, Heatmap showing the relative expression of AP-1 family transcription factors in NCI-H23 cells after treatment by ARS-1620 (1 μM) for 24 h, as indicated by quantitative real-time PCR. *H*, heatmap showing the relative expression of AP-1 family transcription factors in NCI-H23 cells after treatment by BI-2865 (1 μM) for 24 h, as indicated by quantitative real-time PCR. *I*, dose-dependent downregulation of FOS expression in NCI-H23 cells after exposure to various concentrations (0, 10, 20, 50, 100, 200, 500, 1000 nM) of AMG-510 (*left*) or MRTX849 (*right*) for 24 h. *J*, upregulation of UNC5C expression at the mRNA level induced by FOS knockdown in NCI-H23 cells, as indicated by quantitative real-time PCR. *K*, upregulation of UNC5C expression at the protein level induced by FOS knockdown in NCI-H23 cells, as indicated immunoblot analysis. *L*, downregulation of UNC5C expression at the mRNA level induced by FOS overexpression in NCI-H23 cells, as indicated by quantitative real-time PCR. *M*, downregulation of UNC5C expression at the protein level induced by FOS overexpression in NCI-H23 cells, as indicated by immunoblot analysis. *N*, the relative luciferase activity driven by the empty vector, luciferase reporter construct harboring UNC5C promoter fragment with wild-type or mutant FOS binding site upon KRAS KRAS^G12C^ co-transfection. The predicted FOS binding site within UNC5C promoter was mutated from 5′-TGATTCAT-3′ to 5′-AAAAAAAA-3′. *O*, upregulation of UNC5C and downregulation of FOS expression induced by AMG-510 or MRTX849 treatment in NCI-H23 cells, as indicated by immunoblot analysis. *P*, upregulation of UNC5C and downregulation of FOS expression induced by ARS-1620 treatment in NCI-H23 cells, as indicated by immunoblot analysis. *Q*, upregulation of UNC5C and downregulation of FOS expression induced by BI-2865 treatment in NCI-H23 cells, as indicated by immunoblot analysis. *R*, ChIP-qPCR assay for estimating the binding of FOS to the predicted site of human UNC5C promoter in NCI-H23 cells after exposure to KRAS inhibitor. *S*, ChIP-qPCR assay for estimating the binding of FOS to the predicted site of human UNC5C promoter in NCI-H23 cells after KRAS knockdown. ∗*p* < 0.05; ∗∗*p* < 0.01. KD, knockdown; OE, overexpression.

Next, we sought to explore the potential downstream transcription factors of the ERK pathway responsible for the control of UNC5C expression upon KRAS inhibitor treatment. To determine the transcription factors potentially involved in regulating the expression of UNC5C, we initially employed three online databases (FIMO, JASPAR and HumanTFDB) using the promoter sequence of human UNC5C as input to predict transcription factors with a putative binding site within UNC5C promoter (Fig. 4*A*). By intersecting the transcription factors possessing the potential to directly bind to UNC5C promoter predicted by FIMO, JASPAR and HumanTFDB, we identified 51 transcription factors as candidate upstream regulators of UNC5C. Subsequently, we utilized another two online databases identifying regulators of genes by analyzing ChIP-seq datasets, namely ChIPBase v3.0 and hTFtarget, to narrow down the scope of transcription factors of UNC5C (Fig. 4*B*). Notably, FOXA1, FOXA2, FOXP1 and FOS, were recognized as potential regulators of UNC5C across all the 5 online databases. Moreover, to determine the transcription factor involved in the regulation of UNC5C expression in response to KRAS inhibitor treatment, we also determined the overlap of potential transcription factors of UNC5C predicted by FIMO, JASPAR and HumanTFDB and common DEGs induced by KRAS inhibitors (Fig. 4*C*). Accordingly, FOS and FOXP2, were the only two transcription factors significantly changed after KRAS inhibitor treatment. A putative AP-1 binding site of FOS (5′-TGATTCAT-3′, from −1689 to −1682 bp relative to the TSS), with only one base mismatch in comparison to the canonical AP-1 binding sequence 5′-TGA(C/G)TCA-3′ (also known as TPA-responsive element), was recognized within the promoter of human *UNC5C* by JASPAR (Fig. 4*D*). Moreover, the putative binding site of FOS within the *UNC5C* promoter was highly conserved across primates (Fig. 4*E*) and thus might serve as a critical transcription factor binding site and exert a prominent regulatory function on *UNC5C* gene transcription.

Given that FOS was a critical downstream transcription factor of the ERK pathway, we next explored whether FOS was a transcription factor responsible for regulating the expression of UNC5C after KRAS inhibitor exposure. In view of the fact that FOS proteins (FOS, FOSB, FOSL1, FOSL2) heterodimerize with JUN proteins (JUN, JUNB and JUND) to form transcriptionally active AP-1 transcription factor complexes, we determined the expression of classical AP-1 family members in response to KRAS inhibitor treatment (Fig. 4, *F*–*H*). Notably, the expression of some of the AP-1 family members, in particular FOS and FOSL1, were consistently downregulated after treatment with KRAS inhibitors. In addition, KRAS inhibitor-mediated dose-dependent upregulation of *UNC5C* was accompanied by a dose-dependent downregulation of *FOS* expression at the transcriptional level in KRAS-mutant NSCLC (Fig. 4*I*), suggesting a potential inverse correlation between the expression of FOS and UNC5C. Moreover, knockdown of FOS significantly upregulated the expression of UNC5C at the mRNA and protein levels in KRAS-mutant NSCLC (Fig. 4, *J* and *K*). In contrast, overexpression of FOS significantly downregulated the expression of UNC5C at mRNA and protein levels in KRAS-mutant NCI-H23 cells (Fig. 4, *L* and *M*), further implying the involvement of FOS in the transcriptional repression of UNC5C expression. To further validate the involvement of FOS for the regulation of UNC5C expression by oncogenic KRAS and verify the sequence responsible for this regulation, we performed dual luciferase reporter assays after co-transfection of KRAS^G12C^ construct and luciferase reporter construct harboring UNC5C promoter with wild-type or mutant FOS binding site (Fig. 4*N*). As expected, mutation of the putative binding site of FOS within the promoter of UNC5C significantly increased the luciferase activity driven by oncogenic KRAS co-transfection, suggesting that the sequence 5′-TGATTCAT-3′ responsible for the binding of FOS to UNC5C promoter was critical for the transcriptional repression of UNC5C by oncogenic KRAS. Since KRAS inhibitors mediated the upregulation of UNC5C expression and downregulation of the activation of ERK signaling pathway and its downstream AP-1 transcription factor FOS (Fig. 4, *O*–Q), we next designed primer pairs spanning the putative binding site of FOS within the human UNC5C promoter and employed ChIP-quantitative PCR (ChIP-qPCR) to determine the binding of FOS to the promoter of UNC5C in the absence or presence of KRAS inhibitor treatment (Fig. 4*R*). As expected, we observed a significant enrichment of FOS at the promoter of UNC5C in the absence of KRAS inhibitor treatment, indicating that FOS could bind to the putative binding site of the UNC5C promoter. On the contrary, KRAS inhibitor AMG-510 or MRTX849 led to the dissociation of FOS from the *UNC5C* locus (Fig. 4*R*). Given that KRAS inhibition suppressed the activation of FOS, KRAS inhibitor reduced the binding of FOS to the 5′-TGATTCAT-3′ sequence within the *UNC5C* promoter (Fig. 4*R*), which in turn contributed to the overexpression of UNC5C. Similarly, knockdown of KRAS also induced a decrease of the binding of FOS to the putative binding site of the UNC5C promoter (Fig. 4*S*). Collectively, these data indicated that inhibition of oncogenic KRAS depressed the expression of FOS and suppressed the activation of FOS, thereby decreasing the binding of FOS to the sequence 5′-TGATTCAT-3′ within the promoter of UNC5C and thus contributing to the overexpression of UNC5C.

### UNC5C was downregulated in NSCLC and functioned as a tumor suppressor

**Figure 5:**
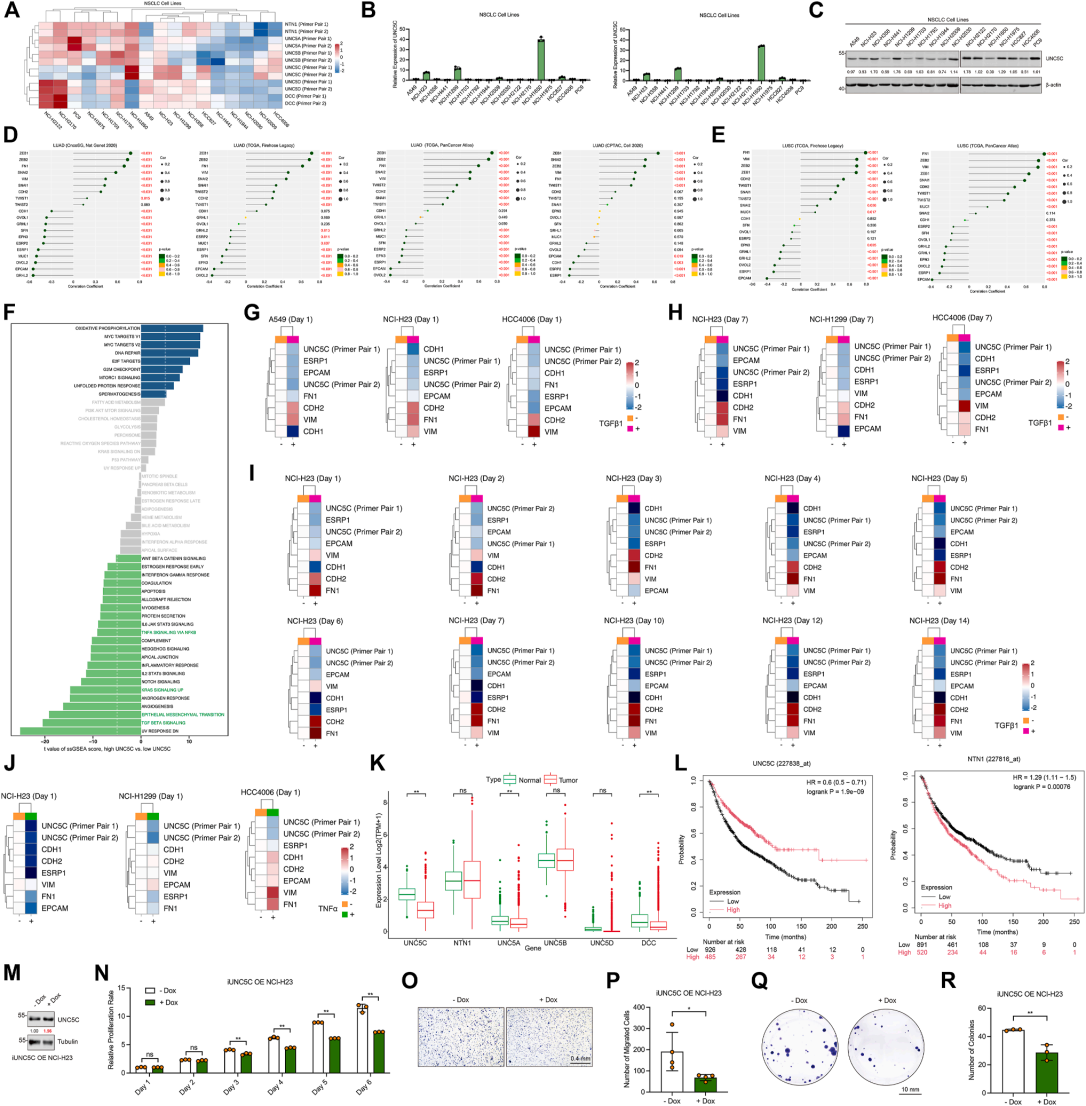
**UNC5C was downregulated in NSCLC and functioned as a tumor suppressor.***A*, heatmap showing the relative expression of *NTN1* and *UNC5A*, *UNC5B*, *UNC5C*, *UNC5D*, *DCC* at mRNA levels in a panel of NSCLC cell lines, as indicated by quantitative real-time PCR. *B*, bar graph showing the relative expression of *UNC5C* at mRNA levels in a panel of NSCLC cell lines, as indicated by quantitative real-time PCR. *C*, the expression of UNC5C at the protein level in a panel of NSCLC cell lines, as indicated by immunoblot analysis. *D*, Lollipop chart showing the correlation between the expression of epithelial or mesenchymal genes and UNC5C in LUAD (OncoSG, Nat Genet 2020), LUAD (TCGA, Firehose Legacy), LUAD (TCGA, PanCancer Atlas), and LUAD (CPTAC, Cell 2020) datasets from the cBioportal (https://www.cbioportal.org/). *E*, Lollipop chart showing the correlation between the expression of epithelial or mesenchymal genes and UNC5C in LUSC (TCGA, Firehose Legacy) and LUSC (TCGA, PanCancer Atlas) datasets from the cBioportal (https://www.cbioportal.org/). *F*, the ssGSEA pathway ranking plot showing the hallmark pathways enriched in UNC5C^high^ in comparison with UNC5C^low^ tumor tissues of NSCLC patients. *G*, heatmap showing the relative expression of epithelial or mesenchymal marker genes in A549 (*left*), NCI-H23 (*middle*) and HCC4006 (*right*) cells after stimulation by TGFβ1 (10 ng/ml) for 1 day, as indicated by quantitative real-time PCR. *H*, heatmap showing the relative expression of epithelial or mesenchymal marker genes in NCI-H23 (*left*), NCI-H1299 (*middle*) and HCC4006 (*right*) cells after stimulation by TGFβ1 (10 ng/ml) for seven consecutive days, as indicated by quantitative real-time PCR. *I*, heatmap showing the relative expression of epithelial or mesenchymal marker genes in NCI-H23 cells after treatment by TGFβ1 (10 ng/ml) for indicated time periods, as indicated by quantitative real-time PCR. *J*, heatmap showing the relative expression of epithelial or mesenchymal marker genes in NCI-H23 (*left*), NCI-H1299 (*middle*) and HCC4006 (*right*) after stimulation by TNFα (40 ng/ml) for 1 day, as indicated by quantitative real-time PCR. *K*, differential expression analysis of DRs of netrin-1 (NTN1), including UNC5A, UNC5B, UNC5C, UNC5D and DCC, as well as NTN1, between NSCLC and noncancerous tissues. *L*, Kaplan-Meier plots showing the correlation between the expression of UNC5C (*left*) or NTN1 (*right*) in tumor tissues and survival probabilities in patients with lung cancer. *M*, immunoblot analysis of UNC5C expression in inducible UNC5C-overexpressing NCI-H23 NSCLC cells in the absence or presence of Dox induction. *N*, CCK-8 assay of inducible UNC5C-overexpressing NCI-H23 NSCLC cells for six consecutive days. *O*, representative photographs of the Transwell migration assay of inducible UNC5C-overexpressing NCI-H23 NSCLC cells. Scale bar: 0.4 mm. *P*, quantitative analysis of the number of migrated cells of inducible UNC5C-overexpressing NCI-H23 NSCLC cells. *Q*, representative photographs of colony formation assay of inducible UNC5C-overexpressing NCI-H23 NSCLC cells. Scale bar: 10 mm. *R*, quantitative analysis of colony formation capacity of inducible UNC5C-overexpressing NCI-H23 NSCLC cells. iUNC5C OE NCI-H23: inducible UNC5C-overexpressing NCI-H23 cells. “- Dox” represents the condition without Dox-induced overexpression of UNC5C, whereas “+ Dox” represents the condition with Dox-induced overexpression of UNC5C. ∗*p* < 0.05; ∗∗*p* < 0.01. See also Figure S7. Dox, doxycycline.

Next, we determined the expression of netrin-1 (*NTN1*) and its DRs (*e*.*g*. *UNC5A*, *UNC5B*, *UNC5C*, *UNC5D*, and *DCC*) at the mRNA level in a panel of human NSCLC cell lines (Fig. 5*A*). Interestingly, UNC5A and UNC5B, as well as UNC5D and DCC, exhibited similar gene expression patterns in human NSCLC cell lines, whereas UNC5C exhibited a distinct gene expression pattern from other DRs of netrin-1. To be specific, UNC5C exhibited heterogeneous expression at the transcriptional level in NSCLC cell lines, with relatively high expression in NCI-H1650, NCI-H1299, NCI-H23, HCC827, NCI-H2009 and HCC4006 cell lines and relatively low expression in NCI-H441, NCI-H1975, NCI-H2030, NCI-H1944, NCI-H2122, NCI-H2170, NCI-H1703, PC9 and NCI-H1792 cell lines (Fig. 5, *A* and *B*). In parallel, we also examined the expression of UNC5C at the protein level in human NSCLC cell lines and found that the protein levels of UNC5C were highly inconsistent with its mRNA levels (Fig. 5*C*). The discrepancy between gene and protein levels of UNC5C suggested the existence of complicated post-transcriptional, translational or post-translational regulatory mechanisms of UNC5C.

We explored the expression pattern of *UNC5C* in tumor tissues of patients with NSCLC. We initially investigated the genes that were positively or negatively correlated with *UNC5C*, separately in LUAD and LUSC (Fig. S7, *A* and *C*). Notably, a positive correlation between the expression of *UNC5C* and mesenchymal signature genes and a negative correlation between the expression of *UNC5C* and epithelial signature genes were observed among both LUAD and LUSC datasets (Fig. S7, *B* and *D*). More specifically, the mesenchymal genes, in particular *ZEB1*, *ZEB2*, *FN1*, and *VIM*, were positively correlated with *UNC5C* expression in tumor tissues of patients with NSCLC at the transcriptional level (Fig. 5, *D* and *E*). In contrast, the epithelial genes, especially *ESRP1* and *EPCAM*, were negatively correlated with *UNC5C* expression (Fig. 5, *D* and *E*). These data indicated that UNC5C was more likely to express in tumor tissues exhibiting a mesenchymal phenotype but not epithelial phenotype in NSCLC. We next explored the hallmark signaling pathways that were correlated with UNC5C expression in tumor tissues of patients with NSCLC (Fig. 5*F*). KRAS signaling was aberrantly enriched in tumor tissues of NSCLC patients exhibiting low UNC5C expression, further suggesting a strong negative correlation between KRAS signaling and UNC5C expression. Notably, UNC5C expression was also inversely correlated with epithelial-to-mesenchymal transition (EMT), as well as TGFβ signaling and TNFα signaling *via* NFκB (53), both of which were crucial pathways for EMT in cancer (Fig. 5*F*). To further validate the correlation between UNC5C expression and EMT, in particular TGFβ signaling and TNFα signaling *via* NFκB, we determined the expression of UNC5C in response to TGFβ1 or TNFα stimulation. Interestingly, UNC5C expression was significantly downregulated in NSCLC cells after TGFβ1 stimulation (Fig. 5*G*) and in cells underwent TGFβ1-induced EMT (Fig. 5, *H* and *I*). Similarly, UNC5C expression was also consistently decreased in response to TNFα treatment in NSCLC cell lines (Fig. 5*J*). These data suggested a complicated regulatory network of UNC5C expression.

Netrin-1 and its receptors exert oncogenic functions in cancer *via* either upregulation of netrin-1 or downregulation of its receptors. To examine the potential functional role of the UNC5 homologue receptor family (UNC5A, UNC5B, UNC5C and UNC5D) and DCC in NSCLC, we evaluated the expression of genes encoding these DRs and their ligand netrin-1 in LUAD and LUSC in comparison with noncancerous tissues (Fig. 5*K*). Interestingly, distinct from other members of DRs, *UNC5C* was the most strongly downregulated DR in NSCLC, while its ligand encoding gene *NTN1* remained largely unchanged. These data indicated that UNC5C, rather than other DRs of netrin-1, was specifically downregulated and thus might function as a tumor suppressor in NSCLC. To determine the potential clinical relevance of UNC5C and its ligand netrin-1, we studied the correlation between the expression of UNC5C or NTN1 in tumor tissues and survival probabilities of patients in lung cancer (Fig. 5*L*). Notably, the high expression of *UNC5C* and low expression of *NTN1* in tumor tissues were correlated with increased survival probabilities, further implying the tumor-suppressive role of UNC5C and tumor-promoting role of NTN1 in lung cancer. Collectively, these results indicated that UNC5C, rather than other DRs, might function as a tumor suppressor in NSCLC. To investigate the potential function of UNC5C in NSCLC, we established NSCLC cells expressing doxycycline (Dox)-inducible UNC5C and determined the role of UNC5C overexpression on cell proliferation, migration and colony formation (Fig. 5*M*). To evaluate the effect of UNC5C on cell proliferation of NSCLC cells, we determined the cell viability and proliferation of NSCLC cells with Dox-inducible UNC5C overexpression with or without Dox induction for six consecutive days (Fig. 5*N*). The results indicated that UNC5C overexpression significantly suppressed the proliferation of NSCLC cells. In parallel, we also explored the effect of UNC5C expression on cell migration ability by transwell assay and found that UNC5C overexpression significantly inhibited the migration of NSCLC cells (Fig. 5, *O* and *P*). In addition, we investigated the role of UNC5C overexpression on colony formation capacity of NSCLC cells (Fig. 5, *Q* and *R*). As expected, overexpression of UNC5C significantly suppressed colony formation capacity of NSCLC cells. These data indicated that UNC5C overexpression inhibited the proliferation, migration and clonogenic capacity of NSCLC cells *in vitro*. Taken together, UNC5C was downregulated in NSCLC and functioned as a potential tumor suppressor.

## Discussion

The discovery of a targetable binding pocket within KRAS^G12C^ protein enables the development of KRAS^G12C^-specific inhibitors and thus heralds a new era of precision medicine for the treatment of KRAS^G12C^-driven NSCLC patients. However, current understanding toward the molecular response of KRAS^G12C^-mutated NSCLC cells to KRAS^G12C^ inhibitors is limited. This study identified UNC5C as a downstream effector of oncogenic KRAS signaling and deciphered the exact underlying molecular mechanism. Oncogenic KRAS-mediated activation of RAF/MEK/ERK cascade, more specifically, its downstream AP-1 transcription factor FOS, played a central role in the transcriptional repression of UNC5C expression in KRAS-driven NSCLC. This work proposed a previously undescribed regulatory mechanism between oncogenic KRAS and conditional tumor suppressor UNC5C. Since OCGs and TSGs execute their cellular and molecular biological functions jointly rather than individually, the interplay and regulatory network between OCGs and TSGs may provide novel insights into the molecular mechanisms by which OCGs and TSGs jointly contribute to cancer initiation and progression. Our findings suggest that oncogenic KRAS-directed UNC5C downregulation probably contributes to tumorigenesis and thus may serve as a promising therapeutic target in oncogenic KRAS-driven NSCLC.

Despite aberrant hypermethylation of UNC5C promoter was observed in COAD and READ, the promoter region of UNC5C was not specifically hypermethylated in other types of cancer, including LUAD and LUSC, and the expression of UNC5C is not correlated with the methylation of its promoter, suggesting the existence of diverse molecular mechanisms mediating the loss or downregulation of UNC5C. In the current study, we uncovered a previously hidden regulatory mechanism of UNC5C expression by oncogenic KRAS in NSCLC. We found that oncogenic KRAS signaling mediated the transcriptional downregulation of UNC5C expression through activation of RAF/MEK/ERK pathway rather than PI3K/AKT/mTOR cascade in NSCLC. More specifically, ERK2, but not ERK1, was responsible for the control of UNC5C expression. Although it is widely recognized that the highly homologous ERK isoforms possess largely overlapping or redundant biological functions in many cellular processes (54), increasing studies have suggested that ERK1 and ERK2 play distinct or specific roles in the regulation of DNA replication and cell proliferation (55), cell migration (56), senescence (57), EMT and gene expression (58) under certain circumstances. In line with these findings, we identified an isoform-specific contribution of ERK2 to the regulation of UNC5C expression. It has been reported that AP-1 transcription factor FOSL1 and EMT were differentially regulated by ERK1 and ERK2 (58). Similarly, we found that AP-1 transcription factor FOS and UNC5C were also differentially regulated by ERK1 and ERK2, further implying the functional difference between ERK2 and ERK1 in regulating gene expression. Our findings deepen the understanding of the functional differences between ERK1 and ERK2.

It is undoubtedly crucial to establish clinical relevance and significance of our findings by validating the upregulation of UNC5C induced by KRAS inhibitors in more clinically relevant settings. However, the lack of relevant studies of samples from animal models or patients (*e*.*g*., pre- and post-treatment biopsies), together with the limited expression of UNC5C in KRAS-mutant cancers, poses an unprecedented challenge for direct validation of UNC5C upregulation by KRAS inhibitor in clinically relevant contexts. Therefore, further studies are still needed to validate the upregulation of UNC5C by KRAS inhibitor and strengthen the translational significance of our findings if more clinical data of tumor biopsies from KRAS-mutant patients collected before and after KRAS inhibitor treatment become publicly available. In addition, it is meaningful to establish the generalizability of the upregulation of UNC5C induced by inhibition of oncogenic KRAS in other KRAS-driven cancers, in particular those with a high frequency of KRAS mutations (*e*.*g*., colorectal cancer and pancreatic cancer). However, the transcriptional induction of UNC5C expression by KRAS inhibitor was largely contingent on basal UNC5C expression in KRAS-driven cancers, underscoring the significance of a comprehensive profiling of baseline gene expression of UNC5C. As a putative tumor suppressor, UNC5C was frequently downregulated or even silenced in many cancers. Given that UNC5C expression was frequently silenced in colorectal cancers due to promoter methylation or loss of heterozygosity (38, 39, 59), KRAS inhibitor treatment alone was unlikely to broadly induce the upregulation of UNC5C in colorectal cancers unless epigenetic or genetic inactivation of UNC5C was reversed. Similarly, in the contexts of complete loss of UNC5C expression, inhibition of KRAS could not restore the expression of UNC5C in other KRAS-driven cancers. Therefore, the molecular mechanisms mediating the complete loss of UNC5C expression in KRAS-mutant cancers other than colorectal cancers remain to be fully elucidated.

The heterogeneous expression of UNC5C in NSCLC cell lines and tumor tissues suggested a complex regulatory network of UNC5C expression. In this study, we observed a notable divergence between mRNA and protein levels of UNC5C, which might be attributed to multiple regulatory mechanisms of UNC5C expression, including post-transcriptional, translational and post-translational controls. For instance, rapid mRNA decay and high translation efficiency may largely limit the mRNA levels of UNC5C while allowing for efficient conversion of mRNA into protein. Similarly, given that the UNC5C protein may be degraded *via* diverse mechanisms (*e*.*g*., ubiquitin-proteasome pathway, autophagy-lysosome pathway and ligand-mediated endocytosis), the complexity and diversity of UNC5C degradation mechanisms across different cellular contexts may also lead to the discrepancy between mRNA and protein levels of UNC5C. In addition, post-translational modification of UNC5C protein (*e*.*g*,. phosphorylation, glycosylation and ubiquitination) may affect its stability, localization and detection, thereby contributing to the inconsistency between mRNA and protein. These factors may individually or jointly contribute to the observed mRNA-protein expression discrepancy and the predominant mechanism may vary across cellular contexts. Therefore, further studies are still needed to dissect the exact mechanisms mediating the discordance between mRNA and protein levels of UNC5C expression. Despite it is generally recognized that the expression of UNC5C is regulated at multiple levels, including transcription, post-transcription and translation, current understanding towards the transcriptional regulatory network of UNC5C in cancer is limited. In this study, we systematically analyzed the potential transcription factors of human UNC5C and identified 51 transcription factors harboring putative binding site within the promoter region of UNC5C. Notably, our data indicated that UNC5C was a direct target of AP-1 transcription factor FOS. Despite the AP-1 binding site 5′-TGATTCAT-3′ identified within the human UNC5C gene promoter was differed from the consensus sequence 5′-TGA(C/G)TCA-3′ by one nucleotide, previous findings have confirmed that the sequence could be bound by AP-1 transcription factor FOS (60). In line with these results, we found that FOS was likely to bind with the AP-1 site within the human UNC5C promoter and thus mediated the transcriptional repression of UNC5C. Although our findings identified FOS as a transcriptional repressor of UNC5C, it requires further research to determine whether other transcription factors predicted in this study were actually involved in the control of UNC5C expression.

The molecular regulatory mechanisms of the expression of the UNC5 homologue family members of DRs may present potential therapeutic targets and opportunities. EMT contributes to tumor initiation, invasion, and metastasis as well as chemotherapy failure. A recent study indicated that UNC5B was highly correlated with EMT scores in human NSCLC and skin cutaneous melanoma (35). In this study, we found that UNC5C expression was positively correlated with EMT signature genes but negatively correlated with epithelial genes in tumor tissues of NSCLC patients. However, further bioinformatics analysis indicated that UNC5C expression exhibited strong negative correlation with EMT signaling pathway in tumor tissues of NSCLC patients. Moreover, the expression of UNC5C was downregulated in response to TGFβ1 or TNFα stimulation, suggesting that the cells integrate extracellular signals and decide the expression of UNC5C. Further studies are still needed to clarify the complicated molecular regulatory mechanisms of UNC5C in NSCLC. In addition, despite netrin-1/UNC5C axis being an alternative therapeutic target for NSCLC, it should be noted that the UNC5C may play a dual role as either tumor suppressor or oncogenic receptor depending on the availability of its ligand. In view of the dependence of UNC5C function on the availability of its ligand netrin-1, the expression of UNC5C may exert divergent effects in cancer as either tumor suppressor or oncogenic receptor, thus making it valuable to determine the expression profile of netrin-1 in cancer.

Our findings concerning the functional roles of UNC5C overexpression on cell proliferation, migration and colony formation in NSCLC supported the tumor suppressive effects of UNC5C protein. Although it is generally recognized that the DRs transduce oncogenic signals upon engagement by their respective ligands but transmit pro-apoptotic signals in the absence of ligand binding (23), the downstream pro-apoptotic pathways of UNC5C remain poorly characterized, particularly in comparison to other UNC5 homologue family members and DCC (26, 61). Moreover, further investigations are required to identify the critical downstream effector pathways regulated by UNC5C protein responsible for its tumor suppressive function. Given that the DRs and their respective ligands exert oncogenic functions in cancer through either loss of the expression of DRs or gain of the expression of ligands, the major challenge in clinical application of signalings mediated by DRs for cancer therapy is to selectively activate their pro-apoptotic signals but not oncogenic signals in cancerous cells. With respect to the bifunctional characteristics of DRs, either simultaneously restoring the expression of the DRs and suppressing the expression of their respective ligands in case of loss of the expression of DRs or blocking the binding of the ligands to DRs in case of gain of the expression of ligands, may be a feasible therapeutic strategy for cancer. Most recently, targeting netrin-1/UNC5B axis has emerged as a promising therapeutic strategy for the treatment of cancer (35, 36). NP137, a humanized neutralizing monoclonal antibody disrupting the interaction between netrin-1 and UNC5B, was under clinical investigation for the treatment of cancer, either alone or in combination with chemotherapy, immunotherapy, multidrug adjuvant therapy or other therapies (NCT02977195, NCT04652076, NCT05546853, NCT05546879, NCT05605496, NCT06150040, NCT06203821). The therapeutic strategies targeting the interaction between UNC5B and its ligand netrin-1, could possibly be extended to other DRs, including UNC5A, UNC5C, UNC5D, and DCC. However, since current understanding of signaling transduction pathways mediated by netrin-1 and its DRs is far from complete, further preclinical and clinical studies are still needed to facilitate the development of targeted therapies harnessing dependence receptor signalings for cancer.

Our findings provided molecular insights into the underlying mechanism of oncogenic KRAS-directed downregulation of conditional tumor suppressor UNC5C, which may be potentially relevant to the initiation and progression of KRAS-driven NSCLC. Nowadays, the DRs and their ligands have received increasing attention in the field of cancer therapy. As a member of UNC5 homologue family DRs, UNC5C possesses opposing functional roles in cancer depending on the availability of its ligand netrin-1 (25). To be specific, the biological role of UNC5C in cancer is context-dependent, varying from pro-apoptotic to anti-apoptosis function. The unique pro-apoptotic properties of DRs in the absence of ligand binding offer alternative therapeutic strategies for cancer patients on the basis of reactivating the expression of DRs or blocking the interaction between DRs and their ligands. Due to the complexity of signaling pathways mediated by UNC5 homolog family DRs, further understanding towards the exact functional role of each member in cancer initiation, progression and metastasis may facilitate the proper harnessing of the dependence receptor signalings for the treatment of cancer. Moreover, novel insights into the regulatory mechanism of expression of DRs may eventually contribute to the discovery of innovative therapeutic targets and strategies.

## Experimental procedures

### Cell culture of human NSCLC cell lines

The human NSCLC cell lines were obtained from the American Type Culture Collection unless otherwise specified. The NCI-H2009 cell line was kindly provided by Prof. Wufan Tao at Fudan University. The human NSCLC cell lines, including A549, NCI-H23, NCI-H358, NCI-H441, NCI-H1299, NCI-H1703, NCI-H1792, NCI-H1944, NCI-H2009, NCI-H2030, NCI-H2122, NCI-H2170, NCI-H1650, NCI-H1975, HCC827, HCC4006 and PC9, were cultured with RPMI 1640 medium containing 10% FBS, 100 U/ml penicillin, and 100 μg/ml streptomycin at 37 °C in a humidified 5% CO_2_ incubator. Then the cell lines were initially genotyped for the authentication of cell identities by short tandem repeat analysis and routinely tested for the absence of *mycoplasma* contaminations by PCR (62).

### Chemicals and reagents

The cells were seeded at a density of 5 × 10^5^ cells per 60 mm cell culture dish and allowed overnight for attachment. For cell treatment, the cells were treated with designated drugs for indicated time periods and the samples were collected for subsequent analysis. The details of chemicals utilized in this study were listed in Table S1. For KRAS inhibitor withdrawal experiments, the cells were pre-treated with either AMG-510 (1 μM) or MRTX849 (100 nM) for 24 h. After removal of drug treatment, the cells were washed three times with cold PBS and cultured in fresh medium without inhibitors. And then the cells were collected at the indicated time points (0 h, 3 h, 6 h, 9 h, 12 h, 24 h, 36 h, 48 h, 72 h, 96 h) after washout.

### RNA-seq

RNA-seq was conducted on NCI-H23 cells after treatment with KRAS inhibitor, either AMG-510 (1 μM) or MRTX849 (100 nM), or vehicle control DMSO (Sigma-Aldrich, Cat. #D2650) for 24 h. Three biological replicates were prepared for each condition for subsequent RNA-seq analysis. Briefly, total RNA was isolated from the cells using PureLink RNA Mini Kit (Invitrogen, Cat. #12183025). After the assessment of RNA quality by the Fragment Analyzer System (Alignment), cDNA libraries were prepared using MGIEasy Fast RNA Library Prep Kit (MGI, Cat. #940–000890–00) from RNA for sequencing on MGISEQ-2000RS platform (MGI Tech Co. Ltd) to generate 50 bp single-end reads. Subsequently, the raw sequencing reads were filtered and trimmed using SOAPnuke (v1.5.6) (63) prior to alignment and the resultant clean reads were then mapped to the human genome GRCh38/hg38 with STAR (v2.5.3a) (64). The quantification of expression levels of genes and transcripts was conducted by RSEM (v1.3.1) (65) and the differential expression analysis was performed using DESeq2 package (v1.4.5). The cutoff criteria for DEGs were |log2 (fold change)| > 1 and adjusted *p* value < 0.01. Gene set enrichment analysis (GSEA) was also performed with OmicShare (66).

### Quantitative real-time PCR

Total RNA was isolated from cells after designated treatments for the indicated time periods using EZ-10 DNAaway RNA Mini-Preps Kit (Sangon Biotech, Cat. #B618133), and cDNA was prepared from purified RNA by HiScript II Q RT SuperMix for qPCR (Vazyme, Cat. #R222–01). Quantitative real-time PCR was performed with Hieff qPCR SYBR Green Master Mix (Low Rox Plus) (YEASEN, Cat. #11202ES08) using QuantStudio three (Applied Biosystems). The relative changes in gene expression were determined by 2^-ΔΔCT^ method with ACTB served as a reference gene. To improve the reliability of the results, the expression of netrin-1 and its DRs, in particular *UNC5C*, was evaluated by two independent primer pairs. The primers used for quantitative real-time PCR were listed in Table S2.

### siRNA transfection

For RNA interference, the cells were transfected following the reverse transfection protocol according to the manufacturer’s instruction. Briefly, 5 μl of 20 μM siRNA was added to 1 ml of Opti-MEM (Gibco, Cat. #31985070) and incubated for 5 min to yield a 100 nM of siRNA after thorough mixing. Subsequently, 10 μl of Lipofectamine RNAiMAX (Invitrogen, Cat. #13778150) was then added to 1 ml of 100 nM siRNA in Opti-MEM, mixed gently and incubated for 20 min. Finally, 3 ml of cells at a density of 1 × 10^5^ cells/ml were incubated with the siRNA for 24 h. The samples were collected for further analysis at 48 h after transfection. The siRNAs utilized in this study were synthesized by GenePharma Co., Ltd (Shanghai, China) and listed in Table S3.

### Generation of mutant KRAS constructs and cell transfection

Human wild-type KRAS (KRAS^WT^) was amplified from human NSCLC cell line harboring wild-type KRAS using primers in Table S4 and cloned into *Bam* HI and *Xho* I sites of LPC vector. Human KRAS mutants, including KRAS^G12A^, KRAS^G12C^, KRAS^G12D^, KRAS^G12R^, KRAS^G12S^, KRAS^G12V^, KRAS^G13C^, KRAS^G13D^, KRAS^Q^^61H^ and KRAS^Q61K^, were generated following standard site-directed mutagenesis procedure using the corresponding primers listed in Table S5. HEK293T cells, obtained from American Type Culture Collection and cultured in DMEM medium supplemented with 10% FBS at 80% confluency, were transiently transfected with empty vector, wild-type or mutant KRAS plasmid using Lipofectamine 2000 (Invitrogen, Cat. #11668019). Cells were collected at 48 h after transfection for subsequent analysis.

### Dual luciferase reporter assay

The human UNC5C promoter region fragment (from −2000 to +115 bp relative to the TSS) with or without mutation of putative FOS binding site predicted by JASPAR was constructed into pGL4.10[luc2]. HEK293T cells were co-transfected with luciferase reporter plasmid pGL4.10[luc2] containing either wild-type (WT) or mutant (MUT) UNC5C promoter fragment, renilla luciferase reporter plasmid pGL4.75[hRluc/CMV] and KRAS^WT^ or KRAS^G12C^ overexpression plasmid using polyethyleneimine (Thermo Fisher Scientific, Cat. #AA4389603). The cell lysates were prepared 48 h after transfection and the luciferase reporter assay was performed using Dual Luciferase Reporter Gene Assay Kit (Beyotime, Cat. #RG027) following the manufacturer’s instructions. Renilla luciferase activity was used as an internal control to normalize transfection efficiency. All experiments were performed in triplicate and repeated at least three independent times.

### Cycloheximide chase assay

CHX chase assay was performed to determine the stability of UNC5C protein upon oncogenic KRAS overexpression or KRAS inhibitor treatment. To determine the stability of UNC5C protein upon KRAS^G12C^ overexpression, HEK293T cells were transiently transfected with KRAS^WT^ or KRAS^G12C^ plasmid. At 48 h after transfection, the cells were cultured for an additional 24 h in the absence or presence of CHX (5 μg/ml). In parallel, to determine the stability of UNC5C protein upon KRAS inhibitor treatment, NCI-H23 cells were pretreated with or without KRAS inhibitor (either AMG-510 or MRTX849) for 24 h. After medium replacement, the cells were repeatedly treated with or without KRAS inhibitor in the absence or presence of CHX (5 μg/ml) as indicated for another 24 h. The protein lysates were collected and subjected to immunoblot analysis.

### Immunoblot analysis

After designated treatments for the indicated time periods, the cells were washed with cold PBS and lysed with RIPA buffer. Equal amounts of total proteins were subjected to immunoblot analysis and the immunoreactive bands were visualized and imaged by Odyssey Clx imaging system (LI-COR Biosciences). The signal intensity of each band was quantified using ImageJ software after background subtraction and normalized to that of α-Tubulin or β-Actin. The number shown below each band (upregulated protein indicated in red, downregulated in green, and unchanged in black) was fold change of band intensity relative to control. The detailed information of primary and secondary antibodies used for immunoblot analysis was listed in Table S6.

### *In vivo* studies

To determine whether KRAS inhibitor could upregulate UNC5C expression *in vivo*, we established cell line-derived xenograft murine models of human KRAS-mutant NSCLC by subcutaneously inoculating 5 × 10^6^ NCI-H23 cells suspended in 50% Matrigel (Corning, Cat. #354234) into both flanks of 6-week-old male NCG mice after 7 days of adaptive feeding. As the tumor volumes reached approximately 100 mm^3^, the tumor-bearing mice were randomized into treatment groups of vehicle control (DMSO), AMG-510 and MRTX849 (5 mice per group). KRAS inhibitor (10 mg/kg body weight) or vehicle (DMSO) was delivered daily for seven consecutive days by oral gavage dissolved in 100 μl of corn oil (MedChemExpress, Cat. #HY-Y1888). After anesthetization by isoflurane inhalation, the mice were sacrificed by cervical dislocation and the tumor tissues were immediately collected for further analysis. The *in vivo* studies were performed in accordance with protocols approved by Animal Ethical Committee of School of Basic Medical Sciences at Fudan University.

### IHC staining and analysis

The freshly isolated tumor tissues were subjected to IHC analysis of UNC5C expression. Semiquantitative analysis of UNC5C expression from IHC staining of xenografts tumors was assessed with H-score using IHC profiler of ImageJ software, which was calculated by the following formula: H-score = (percentage of weak intensity × 1) + (percentage of moderate intensity × 2) + (percentage of strong intensity × 3). The H-score for UNC5C IHC staining of each tumor was calculated by the average of three random fields. The detailed information of primary antibody against UNC5C used for IHC staining was presented in Table S7.

### Bioinformatics analysis of potential transcription factors of UNC5C

Three online databases, including FIMO (67), JASPAR (68), and HumanTFDB (69), were initially employed to predict the transcription factors that possess putative binding sites within the gene promoter of human UNC5C. The following criteria were utilized to filter the potential transcription factors: *p*-value was < 0.0001 for FIMO; the relative profile score threshold was > 90% for JASPAR; and the score was ≥ 10 with q-value < 0.05 for HumanTFDB. Subsequently, the other two databases, including ChIPBase v3.0 (70) and hTFtarget (71), were also applied to provide more reliable prediction of transcription factors of UNC5C on the basis of analyzing ChIP-seq datasets.

### Prediction of potential binding sites of FOS within the UNC5C promoter by JASPAR

The prediction of putative transcription factor binding site of human AP-1 transcription factor FOS within the promoter region of human UNC5C (upstream = 2000, TSS, downstream = 99) was performed by the open-access database JASPAR (68) with the relative profile score threshold set to 90%.

### Conservation analysis of putative binding site of FOS within UNC5C promoter

The DNA sequences of putative binding site of FOS within UNC5C promoter were retrieved from UCSC genome browser. The graphical representation of nucleic acid multiple sequence alignments of putative binding site of FOS within the UNC5C promoter across primates was created by WebLogo (https://weblogo.berkeley.edu/logo.cgi).

### Single sample gene set enrichment analysis (ssGSEA)

The ssGSEA pathway ranking plot was generated using transcriptome profiling data of tumor samples from the integrated TCGA-LUAD and TCGA-LUSC dataset. The gene expression values were Transcripts Per Million (TPM) transformed as log2(TPM + 1), and ssGSEA was performed using the R package GSVA (v1.52.3) for Hallmark pathways obtained from MSigDB (v7.5.1). The samples were divided into UNC5C^high^ and UNC5C^low^ expression groups using the average gene expression level of UNC5C as cutoff. Differential pathway activity between the two groups was analyzed using the limma package (v3.60.6). The t-values from the limma analysis were visualized using ggplot2 (v3.5.1) and ggthemes (v5.1.0).

### ChIP-qPCR

ChIP assay was performed with cross-linked chromatin from NCI-H23 cells after designated treatment using SimpleChIP Plus Enzymatic Chromatin IP Kit (Cell Signaling Technology, Cat. #9005) following the manufacturer’s instruction with minor modification. Briefly, after cross-linking, the chromatin was digested into fragments with micrococcal nuclease (Cell Signaling Technology, Cat. #10011) and precipitated using isotype control (Cell Signaling Technology, Cat. #2729) or anti-FOS antibody (Cell Signaling Technology, Cat. #2250). Subsequently, the antibody-protein-chromatin complexes were captured with Protein A/G magnetic beads (MedChemExpress, Cat. #HY-K0202). The precipitated DNA was subjected to qPCR using primer pairs designed to amplify the FOS binding locus of UNC5C promoter (from −1699 to −1542 bp relative to the TSS). The primers used for amplifying ChIP products were provided in Table S8.

### Generation of Dox-inducible UNC5C-overexpressing NSCLC cells

The Dox-inducible UNC5C-overexpressing NSCLC cells were generated using a Tet-On lentiviral system. Briefly, the coding sequence of UNC5C was subcloned into the pLVX-TetOne-Puro lentiviral vector (Takara, Cat. #631849) to construct the recombinant plasmid overexpressing UNC5C. Subsequently, HEK293T cells were co-transfected with the recombinant plasmid, the packaging plasmid psPAX2 (Addgene, Cat. #12260) and the envelop plasmid pMD2.G (Addgene, Cat. #12259) using polyethyleneimine (Thermo Fisher Scientific, Cat. #AA4389603) to produce lentivirus. The viral supernatants harvested at 48 h after transfection were used to infect NCI-H23 cells for 48 h. And then the NCI-H23 cells transduced by the lentivirus were selected with 1 μg/ml puromycin for 10 to 14 days to obtain polyclonal cell line (termed iUNC5C OE NCI-H23). To induce UNC5C overexpression, the iUNC5C OE NCI-H23 cells were treated with 1 μg/ml Dox for 48 h and the overexpression of UNC5C was confirmed by immunoblot analysis.

### Cell viability and proliferation assay

The effects of UNC5C overexpression on cell proliferation and viability of NSCLC cells were determined using cell counting kit-8 (CCK-8). The cells with or without Dox induction were seeded into 96-well plates at a density of 1 × 10^3^ cells/well and cultured overnight for adherence. Subsequently, the cells were cultured continuously for the indicated time periods. And 10 μl of CCK-8 solution was added to each well and incubated for 1 h at 37 °C in a 5% CO_2_ incubator. The absorbance at 450 nm was measured using a microplate reader (Molecular Devices, SpectraMax i3x).

### Transwell migration assay

Transwell assay was performed to evaluate the effects of UNC5C overexpression on cell migration capacity of NSCLC cells using 24-well Transwell chambers with 8 μm pore size polycarbonate membranes. Briefly, the cells with or without Dox induction were harvested and resuspended in serum-free medium. A total of 1 × 10^5^ cells in 100 μl serum-free medium were seeded to the upper chamber, while 600 μl of complete medium supplemented with 10% FBS was added to the lower chamber. After incubation at 37 °C in a 5% CO_2_ incubator for 24 h, cells that had migrated through the membranes were fixed with 4% paraformaldehyde for 20 min and stained with 0.1% crystal violet for 15 min followed by photographed under an inverted microscope. The numbers of migrated cells in five random fields were counted for quantitative analysis to assess the cell migration capacity.

### Clonogenic assay

Colony formation assay was performed to determine the effects of UNC5C overexpression on clonogenicity of NSCLC cells. In brief, the cells with or without Dox induction were seeded into six-well plates at a density of 500 cells/well and cultured continuously in complete medium for 14 to 21 days at 37 °C in a 5% CO_2_ incubator until visible colonies formed. Subsequently, the colonies formed were washed twice with PBS, fixed with 4% paraformaldehyde for 15 min and then stained with 0.1% crystal violet solution for 20 min at room temperature. Colony counting was conducted after the plates were rinsed with distilled water, air-dried and photographed.

### Statistical analysis

Statistical analysis was performed with Graphpad Prism (10.0.2). All data were presented as mean ± standard deviation (SD). Student’s t tests were used to calculate *p* values and test statistical significance. *p* values less than 0.05 were considered statistically significant, unless otherwise specified. The association between UNC5C expression and EMT marker genes in LUAD and LUSC was calculated using the Pearson correlation coefficients. Kaplan-Meier survival curves showing the correlation between the expression of UNC5C or NTN1 and survival probabilities in lung cancer were generated by Kaplan-Meier plotter (72), and the statistical differences between groups were determined using log-rank tests.

## Data availability

The RNA-seq data have been deposited in the Gene Expression Omnibus database (73) under the accession code GSE293400. Any additional information of this study are available from the lead contact (shaofeiwang@fudan.edu.cn) upon request.

## Supporting information

This article contains supporting information (39, 74, 75, 76, 77, 78, 79, 80, 81, 82, 83, 84, 85, 86, 87, 88, 89, 90).

## Conflict of interest

The authors declare that they have no conflicts of interest with the contents of this article.
