# Supplementary material for: Oncogenic KRAS represses the dependence receptor UNC5C via ERK2-FOS signaling in non-small cell lung cancer
Source: J Biol Chem. 2026 Jun 19;302(8):113262. doi: 10.1016/j.jbc.2026.113262 (PMC13444284; doi:10.1016/j.jbc.2026.113262)
Supplement: Supplementary file 1 [file mmc1.pdf]

Supporting Information for

Oncogenic KRAS Represses the Dependence Receptor UNC5C via ERK2-FOS Signaling in Non-Small Cell Lung Cancer

Deyu Wang[1], Man Xiao[1,2], Kaicheng Zhou[3], Peng Yang[1], Xiting Huang[3], Runze Miao[1], Jiajun Fan[3,*], Xuyao Zhang[3,*], Shaofei Wang[1,*]

[1]Department of Cellular and Genetic Medicine, School of Basic Medical Sciences, Fudan University, Shanghai 200032, P.R. China

[2]Molecular Diagnostic Laboratory, Department of Clinical Laboratory, Shanghai Children's Hospital, School of Medicine, Shanghai Jiao Tong University, Shanghai 200040, P.R. China

[3]Department of Biological Medicines, School of Pharmaceutical Sciences, Shanghai Engineering Research Center of Immunotherapeutics, Fudan University, Shanghai 201203, P.R. China

***Correspondence to:**

Jiajun Fan, Department of Biological Medicines, School of Pharmaceutical Sciences, Shanghai Engineering Research Center of Immunotherapeutics, Fudan University, Shanghai 201203, China

E-mail: jiajunfan12@fudan.edu.cn

Xuyao Zhang, Department of Biological Medicines, School of Pharmaceutical Sciences, Shanghai Engineering Research Center of Immunotherapeutics, Fudan University, Shanghai 201203, China

E-mail: xuyaozhang@fudan.edu.cn

Shaofei Wang, Department of Cellular and Genetic Medicine, School of Basic Medical Sciences, Fudan University, Shanghai, 200032, P.R. China

E-mail: shaofeiwang@fudan.edu.cn

Table S1. Chemicals and recombinant proteins used in this study.

Chemicals & Recombinant Proteins	Category	Concentration	Source	Catalog Number
Sotorasib (AMG-510)	$KRAS^{G12C}$ inhibitor	0, 10, 20, 50, 100, 200, 500, 1000 nM	MedChemExpress	Cat. #HY-114277
Adagrasib (MRTX849)	$KRAS^{G12C}$ inhibitor	0, 10, 20, 50, 100, 200, 500, 1000 nM	MedChemExpress	Cat. #HY-130149
ARS-1620	$KRAS^{G12C}$ inhibitor	0, 10, 20, 50, 100, 200, 500, 1000 nM	MedChemExpress	Cat. #HY-U00418
BI-2865	pan-KRAS inhibitor	0, 10, 20, 50, 100, 200, 500, 1000 nM	MedChemExpress	Cat. #HY-153724
LY3009120	pan-RAF inhibitor	10 μM	MedChemExpress	Cat. #HY-12558
U0126	MEK1/2 & ERK1/2 inhibitor	10 μM	MedChemExpress	Cat. #HY-12031
PD98059	MEK1/2 & ERK1/2 inhibitor	20 μM	MedChemExpress	Cat. #HY-12028
LY294002	PI3K inhibitor	10 μM	MedChemExpress	Cat. #HY-10108
Triciribine (TCN)	AKT inhibitor	1 μM	Selleckchem	Cat. #S1117
MK-2206	AKT inhibitor	2 μM	Selleckchem	Cat. #S1078
Torin2	mTOR inhibitor	100 nM	MedChemExpress	Cat. #13002
Cycloheximide (CHX)	Protein synthesis inhibitor	5 μg/mL	Sigma-Aldrich	Cat. #C7689
Doxycycline (Dox)	Activator of inducible gene expression system	1 μg/mL	MedChemExpress	Cat. # HY-N0565B
Recombinant human TGFβ1	EMT inducer	10 ng/mL	Novoprotein	Cat. #CA59
Recombinant human TNFα	Cytokine	40 ng/mL	Novoprotein	Cat. #C008

Table S2. The primers used for quantitative real-time PCR.

Symbol	Gene ID	Forward Primer	Reverse Primer	Amplicon Size (bp)	Source
UNC5C	8633	TGCAAATGCTCGTGCTACCT	TGTGGTCCTTCTGATGAACCC	258	(1)
UNC5C	8633	ACCTGTACTGTAAAGCAAGCC	GGACAATGAGACCGGAAGTTT	120	PrimerBank
NTN1	9423	CTGAAGACTGCGATTCCTACTG	TGGATCTGGACGGCATAGT	94	(2)
NTN1	9423	ACAACCCGCACAACCTGAC	GGGACAGTGTGAGCGTGAC	78	PrimerBank
UNC5A	90249	CTGCGCCTCAAAAAGCAGTC	CTGGCAGTAGTAGAGGTGGGA	90	PrimerBank
UNC5A	90249	TCTACCTCACGCTGCACAAGCC	ACAGTGGTCCATAGCCAGGATG	137	OriGene
UNC5B	219699	ACTGCCGTGACTTCGACAC	GCCTTGCCGTCTTAAAGTTGA	87	PrimerBank
UNC5B	219699	GAGCCGAAACCGCTAATG	CTGCCACTCCAAATGTGATAGA	137	(3)
UNC5D	137970	AAGCCCTTCCCGAATCCATC	GTGCAATAGGGTTGCTCTTGATA	99	PrimerBank
UNC5D	137970	CAAGAGCAACCCTATTGCACT	CTCGTTCTGATGGACCCACTC	91	PrimerBank
DCC	1630	GACTTTACCAATGTGAGGCATCT	GGTCCTGCTACTGCAACTTTT	79	PrimerBank
DCC	1630	GAGACACAGTGCTACTCAAGTG	GGAGTCAGGTCTTGTTGGTTCTT	85	PrimerBank
KRAS	3845	TGTTCACAAAGGTTTTGTCTCC	CCTTATAATAGTTTCCATTGCCTT G	127	(4)
AKT1	207	TCCTCCTCAAGAATGATGGCA	GTGCGTTCGATGACAGTGGT	181	PrimerBank
AKT2	208	CATCCTCATGGAAGAGATCCGC	GAGGAAGAACCTGTGCTCCATG	148	OriGene
AKT3	10000	AATGGACAGAAGCTATCCAGGC	TGATGGGTTGTAGAGGCATCC	130	PrimerBank
ARAF	369	CCTGGCGTTCTGTGACTTCTG	CGGTTGGTACTCATGTCAACAC	129	PrimerBank
BRAF	673	TGGGGAACGGAACTGATTTTTC	TTTTGTGGTGACTTGGGGTTG	154	PrimerBank
CRAF	5894	GGGAGCTTGGAAGACGATCAG	ACACGGATAGTGTTGCTTGTC	165	PrimerBank
MEK1	5604	CAGAAGAAGCTGGAGGAGCTAG	CCATCGCTGTAGAACGCACCAT	311	(5)
MEK2	5605	CGAGGCAAACCTGGTGGACCT	CCGTAGAAGCCCACGATGTAC	315	(5)
ERK1	5595	TCAGACTCCAAAGCCCTTGACCT	AAGCGTGCTGTCTCCTGGAAGAT	218	(5)
ERK2	5594	ATGCTGACTCCAAAGCTCTGGACT	TCTGAGCCCTTGTCCTGACAAAT T	265	(5)
ETV1	2115	CTGAACCCTGTAACTCCTTTCC	AGACATCTGGCGTTGGTACATA	77	PrimerBank
ETV4	2118	GATGAAAGCCGGATACTTGGAC	TTCGCGCAAGCTCCCATTT	79	PrimerBank
ETV5	2119	CAGTCAACTTCAAGAGGCTTGG	TGCTCATGGCTACAAGACGAC	168	PrimerBank

MYC	4609	GTCAAGAGGCGAACACACAAC	TTGGACGGACAGGATGTATGC	162	PrimerBank
FOS	2353	CCGGGGATAGCCTCTCTTACT	CCAGGTCCGTGCAGAAGTC	93	PrimerBank
FOSL1	8061	CAGGCGGAGACTGACAAACTG	TCCTTCCGGGATTTTGCAGAT	132	PrimerBank
EGR1	1958	GGTCAGTGGCCTAGTGAGC	GTGCCGCTGAGTAAATGGGA	149	PrimerBank
IER3	8870	CAGCCGCAGGGTTCTCTAC	GATCTGGCAGAAGACGATGGT	112	PrimerBank
MAFF	23764	CTGTCGGACGAGGCGCTGATG	AGCCACGGTTTTTGAGTGTGCG	121	PrimerBank
DUSP4	1846	CCCACAGAGCAGTATTAGGCTGA AG	CAGCGTGGATGAGCAACTGAA	99	(6)
DUSP5	1847	TGTCGTCCTCACCTCGCTA	GGGCTCTCTCACTCTCAATCTTC	152	Primer Bank
DUSP6	1848	CGACTGGAACGAGAATACGG	GGAGAACTCGGCTTGGAACT	124	(7)
SPRY2	10253	CCTACTGTCGTCCCAAGACCT	GGGGCTCGTGCAGAAGAAT	143	PrimerBank
SPRY4	81848	TCTGACCAACGGCTCTTAGAC	GTGCCATAGTTGACCAGAGTC	275	PrimerBank
SPRED1	161742	GAGGGAGTGGACTAAGCAGC	CCTCTATCAAAAGCCCTAGCATC	241	PrimerBank
SPRED2	200734	TATATTGTGCGTGTCAAGGCTG	GGGGTGCATGACCTTACAGA	114	PrimerBank
PHLDA1	22822	GAAGATGGCCCATTCAAAAGCG	GAGGAGGCTAACACGCAGG	188	PrimerBank
CCND1	595	GCTGCGAAGTGGAAACCATC	CCTCCTTCTGCACACATTTGAA	135	PrimerBank
JUN	3725	TCCAAGTGCCGAAAAAGGAAG	CGAGTTCTGAGCTTTCAAGGT	78	PrimerBank
JUNB	3726	ACGACTCATACACAGCTACGG	GCTCGGTTTCAGGAGTTTGTAGT	80	PrimerBank
JUND	3727	TCATCATCCAGTCCAACGGG	TTCTGCTTGTGTAAATCCTCCAG	136	PrimerBank
FOSB	2354	GCTGCAAGATCCCCTACGAAG	ACGAAGAAGTGTACGAAGGGTT	249	PrimerBank
FOSL2	2355	CAGAAATTCCGGGTAGATATGCC	GGTATGGGTTGGACATGGAGG	130	PrimerBank
CDH1	999	CGAGAGCTACACGTTCACGG	GGGTGTCGAGGGAAAAATAGG	119	PrimerBank
CDH2	1000	AGCCAACCTTAACTGAGGAGT	GGCAAGTTGATTGGAGGGATG	136	PrimerBank
EPCAM	4072	AATCGTCAATGCCAGTGTACTT	TCTCATCGCAGTCAGGATCATAA	178	PrimerBank
ESRP1	54845	GCCAAGCTAGGCTCGGATG	CAGTCCTCCGTCAGTTCCAAC	131	PrimerBank
VIM	7431	GACGCCATCAACACCGAGTT	CTTTGTCGTTGGTTAGCTGGT	238	PrimerBank
FN1	2335	CGGTGGCTGTCAGTCAAAG	AAACCTCGGCTTCCTCCATAA	130	PrimerBank
ACTB	60	CATGTACGTTGCTATCCAGGC	CTCCTTAATGTCACGCACGAT	250	(8)

Table S3. The siRNA oligonucleotides used in this study.

Name	Target Gene	Gene ID	Target Sequence	Source
Non-targeting control	/	/	UGGUUUACAUGUUGUGUGA	GenePharma Co., Ltd (9)
siKRAS #1	KRAS	3845	GGAGGGCUUUCUUUGUGUA	GenePharma Co., Ltd (9)
siKRAS #2	KRAS	3845	GAAGUUAUGGAAUUCCUUU	GenePharma Co., Ltd (9)
siKRAS^{WT}	Wild-type KRAS	3845	GUUGGAGCUGGUGGCGUAG	GenePharma Co., Ltd (10)
siKRAS^{G12C}	KRAS^{G12C}	3845	GUUGGAGCUUGUGGCGUAG	GenePharma Co., Ltd (10)
siKRAS^{G12V}	KRAS^{G12V}	3845	GUUGGAGCUGUUGGCGUAG	GenePharma Co., Ltd (10)
siAKT1	AKT1	207	GACAAGGACGGGCACAUUA	GenePharma Co., Ltd
siAKT2	AKT2	208	GCAAAGAGGGCAUCAGUGA	GenePharma Co., Ltd
siAKT3	AKT3	10000	GCUCAUUCAUAGGAUAUAA	GenePharma Co., Ltd
siARAF	ARAF	369	ACCGAGAUCUCAAGUCUAA	GenePharma Co., Ltd (11, 12)
siBRAF	BRAF	673	CAUGAAGACCUCACAGUAA	GenePharma Co., Ltd (12)
siCRAF	CRAF	5894	GCACGGAGAUGUUGCAGUA	GenePharma Co., Ltd (11)
siMEK1/2	MEK1/2	5604/5605	CAGAAGAAGCUGGAGGAGC	GenePharma Co., Ltd (13)
siERK1	ERK1	5595	GCCAUGAGAGAUGUCUACA	GenePharma Co., Ltd (14)
siERK2	ERK2	5594	CCAAAGCUCUGGACUUAUU	GenePharma Co., Ltd (15)
siFOS	FOS	2353	GGGAUAGCCUCUCUUACUA	GenePharma Co., Ltd (16)

Table S4. The primers used for gene cloning of human wild-type KRAS.

Gene Symbol	Forward Primer	Reverse Primer
KRAS	CGCGGATCCGCGATGACTGAATATAAACTTGTGGTAGTTGGA	CCGCTCGAGCGGTTACATTATAATGCATTTTTTAATTTTCAC

Table S5. The primers used for site-directed mutagenesis of human wild-type KRAS.

KRAS Mutation	Nucleotide Change	Forward Primer	Reverse Primer
KRAS^{G12A}	GGT>GCT	GTTGGAGCTGCTGGCGTAGGCAAGAGTGCCTTGACG	GCCTACGCCAGCAGCTCCAACTACCACAAGTTTATA
KRAS^{G12C}	GGT>TGT	AGTTGGAGCTTGTGGCGTAGGCAAGAGTGCCTTGAC	CCTACGCCACAAGCTCCAACTACCACAAGTTTATAT
KRAS^{G12D}	GGT>GAT	GTTGGAGCTGATGGCGTAGGCAAGAGTGCCTTGACG	GCCTACGCCATCAGCTCCAACTACCACAAGTTTATA
KRAS^{G12R}	GGT>CGT	AGTTGGAGCTCGTGGCGTAGGCAAGAGTGCCTTGAC	CCTACGCCACGAGCTCCAACTACCACAAGTTTATAT
KRAS^{G12S}	GGT>AGT	AGTTGGAGCTAGTGGCGTAGGCAAGAGTGCCTTGAC	CCTACGCCACTAGCTCCAACTACCACAAGTTTATAT
KRAS^{G12V}	GGT>GTT	GTTGGAGCTGTTGGCGTAGGCAAGAGTGCCTTGACG	GCCTACGCCAACAGCTCCAACTACCACAAGTTTATA
KRAS^{G13C}	GGC>TGC	TGGAGCTGGTTGCGTAGGCAAGAGTGCCTTGACGAT	TTGCCTACGCAACCAGCTCCAACTACCACAAGTTTA
KRAS^{G13D}	GGC>GAC	GGAGCTGGTGACGTAGGCAAGAGTGCCTTGACGATA	CTTGCCTACGTCACCAGCTCCAACTACCACAAGTTTA
KRAS^{Q61H}	CAA>CAC	CAGCAGGTCACGAGGAGTACAGTGCAATGAGGGACC	TGTACTCCTCGTGACCTGCTGTGTCGAGAATATCCA
KRAS^{Q61K}	CAA>AAA	CACAGCAGGTAAAGAGGAGTACAGTGCAATGAGGGA	TACTCCTCTTTACCTGCTGTGTCGAGAATATCCAAG

Table S6. Primary and secondary antibodies for immunoblot analysis.

Antibodies	Dilution	Source	Catalog Number
UNC5C-Specific Polyclonal Antibody	1:1000	Proteintech	Cat. #20240-1-AP
Anti-KRAS Antibody	1:1000	Abcam	Cat. Ab275876
Phospho-Akt (Ser473) (D9E) XP® Rabbit mAb	1:1000	Cell Signaling Technology	Cat. #4060
Akt (pan) (C67E7) Rabbit mAb	1:1000	Cell Signaling Technology	Cat. #4691
Phospho-p44/42 MAPK (Erk1/2) (Thr202/Tyr204) Antibody	1:1000	Cell Signaling Technology	Cat. #9101
p44/42 MAPK (Erk1/2) Antibody	1:1000	Cell Signaling Technology	Cat. #9102
Phospho-c-Fos (Ser32) (D82C12) XP® Rabbit mAb	1:1000	Cell Signaling Technology	Cat. #5348
c-Fos Antibody	1:1000	Cell Signaling Technology	Cat. #4384
Beta Actin Monoclonal Antibody	1:5000	Proteintech	Cat. # 66009-1-Ig
Alpha Tubulin Monoclonal Antibody	1:5000	Proteintech	Cat. # 66031-1-Ig
IRDye® 800CW Goat anti-Rabbit IgG Secondary Antibody	1:20000	LI-COR	Cat. #926-32211
IRDye® 680RD Goat Anti-Mouse IgG Secondary Antibody	1:20000	LI-COR	Cat. #926-68070

Table S7. Primary antibodies for immunohistochemistry staining and analysis.

Antibodies	Dilution	Source	Catalog Number
UNC5C-Specific Polyclonal antibody	1:200	Proteintech	Cat. #20240-1-AP

Table S8. The primers used for ChIP-qPCR.

Gene Name	Location from TSS	Forward Primer	Reverse Primer
UNC5C	-1699 to -1542	CGGATCTCCCTGATTCATCCGAG	CCCTGGGTAAGTCAGGTAACCTG

Supplementary Figures and Figure Legends

Figure S1. UNC5C was a potential downstream effector of oncogenic KRAS signaling, related to Figure 1.

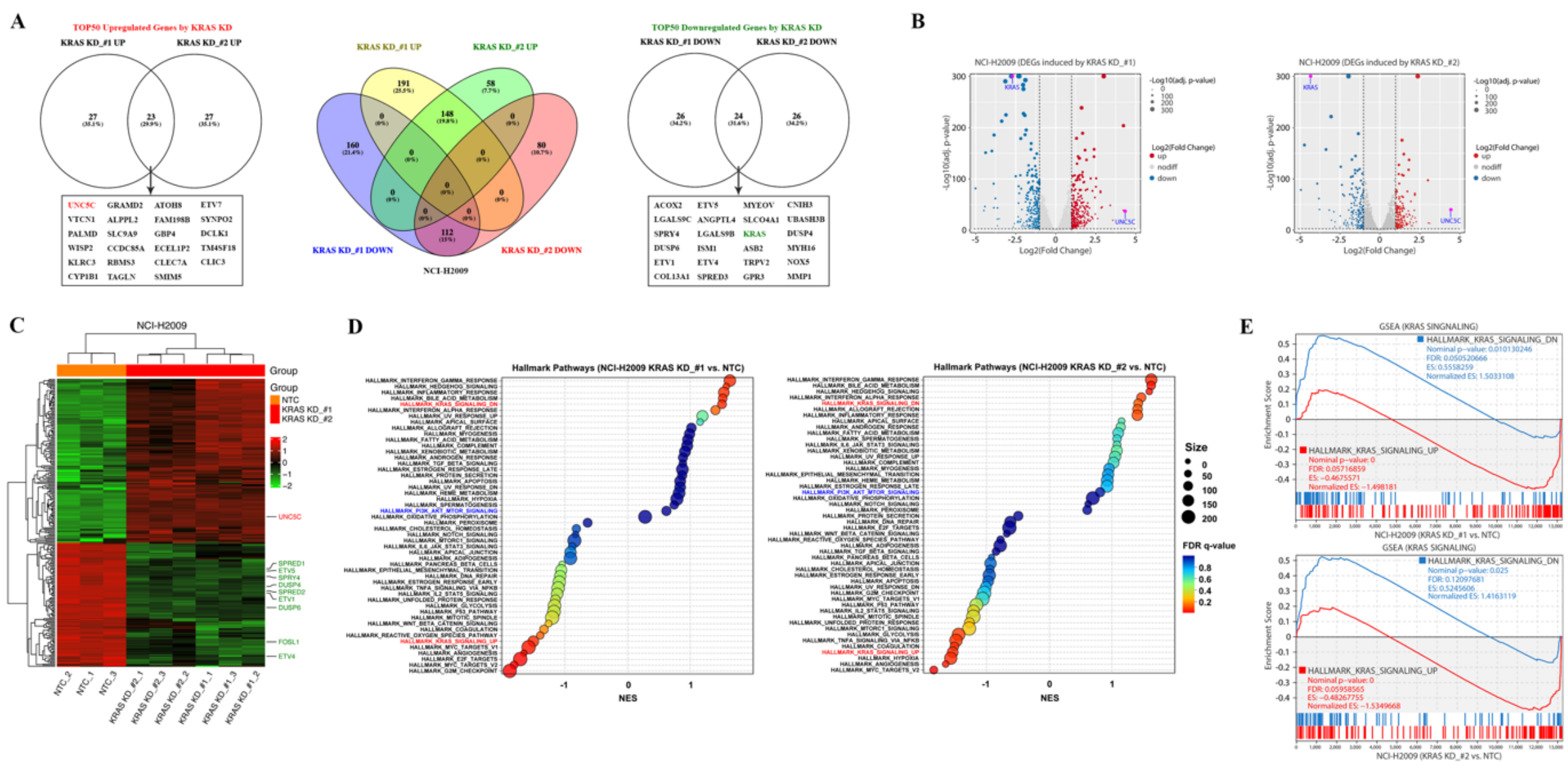

(A) Middle panel: Venn diagram illustrating the common DEGs, including both upregulated and downregulated genes, induced by KRAS knockdown in $KRAS^{G12A}$-mutant NCI-H2009 cells, as indicated by RNA-seq. Left panel: Venn diagram illustrating the overlap of TOP50 upregulated genes after KRAS knockdown by each individual siRNA in NCI-H2009 cells. Right panel: Venn diagram illustrating the overlap of TOP50 downregulated genes after KRAS knockdown by each individual siRNA in NCI-H2009 cells. (B) Volcano plots showing the common DEGs, in particular *UNC5C* and *KRAS*, in $KRAS^{G12A}$-mutant NCI-H2009 cells after KRAS knockdown, as indicated by RNA-seq. (C) Heatmap illustrating the expression of DEGs, in particular *UNC5C* and ERK-dependent genes, in $KRAS^{G12A}$-mutant NCI-H2009 cells after KRAS knockdown, as indicated by RNA-seq. (D) Hallmark pathways from gene set enrichment analysis (GSEA) of genes induced by KRAS knockdown in $KRAS^{G12A}$-mutant NCI-H2009 cells, as indicated by RNA-seq. (E) GSEA analysis of KRAS Signaling Up and KRAS Signaling DOWN signatures in $KRAS^{G12A}$-mutant NCI-H2009 cells in response to KRAS knockdown, as indicated by RNA-seq. Normalized enrichment score (NES) of KRAS Signaling UP and KRAS Signaling DOWN signatures were indicated in red and blue color, respectively. DEGs: differentially expressed genes.

Figure S2. KRAS inhibitor induced dose-dependent upregulation of UNC5C in KRAS-mutant cell lines, related to Figure 1.

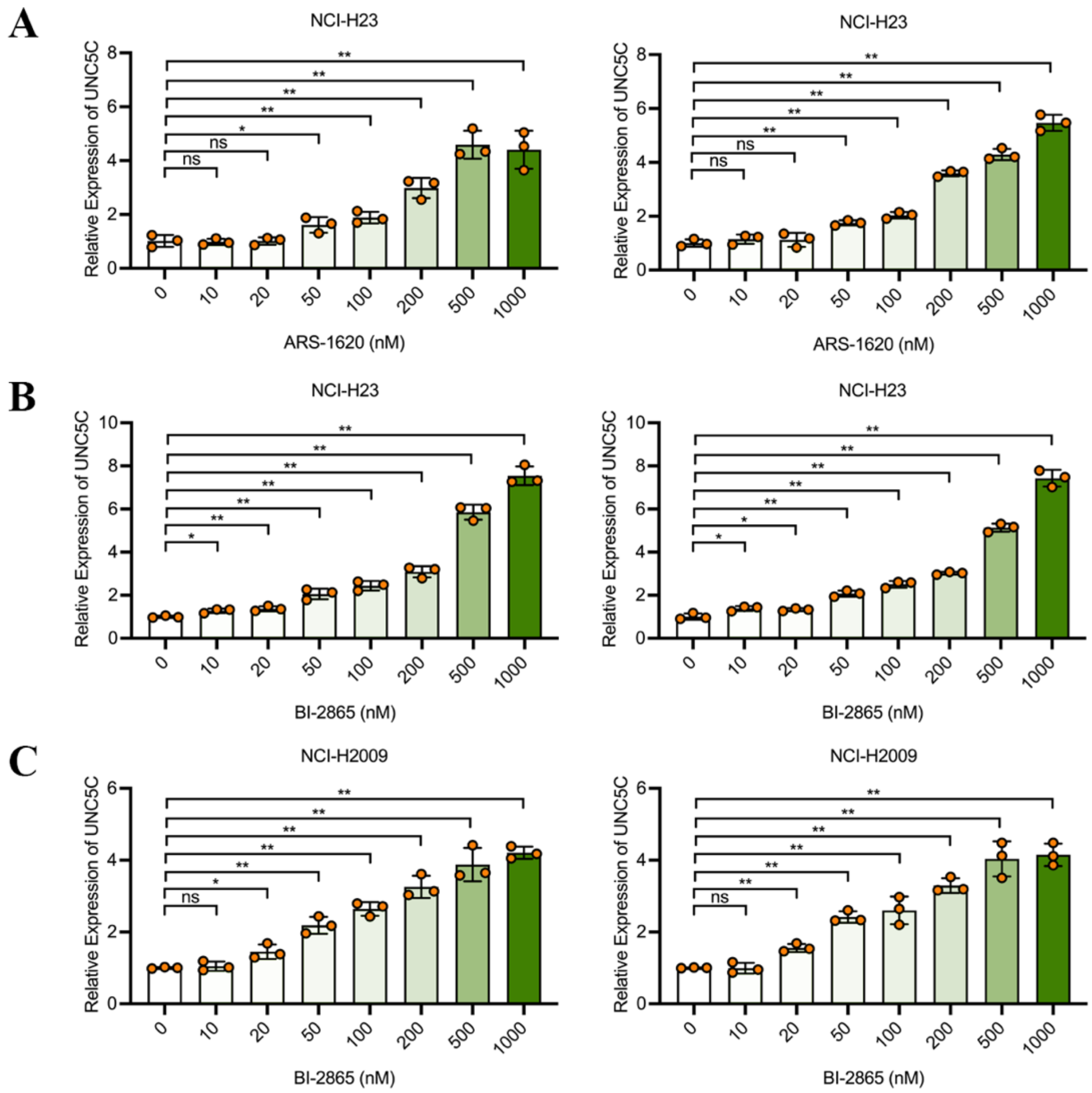

(A) The relative expression of *UNC5C* after KRAS^{G12C}-mutant NCI-H23 cells were treated by various concentrations (0, 10, 20, 50, 100, 200, 500, 1000 nM) of KRAS^{G12C}-specific inhibitor ARS-1620, as assessed by quantitative real-time PCR. (B) The relative expression of *UNC5C* after KRAS^{G12C}-mutant NCI-H23 cells were treated by various concentrations (0, 10, 20, 50, 100, 200, 500, 1000 nM) of pan-KRAS inhibitor BI-2865, as assessed by quantitative real-time PCR. (C) The relative expression of *UNC5C* after KRAS^{G12A}-mutant NCI-H2009 cells were treated by various concentrations (0, 10, 20, 50, 100, 200, 500, 1000 nM) of pan-KRAS inhibitor BI-2865, as assessed by quantitative real-time PCR. *P<0.05; **P<0.01.

Figure S3. KRAS inhibitor failed to induce upregulation of UNC5C in KRAS-mutant cell lines exhibiting undetectable UNC5C expression at mRNA levels, related to Figure 2.

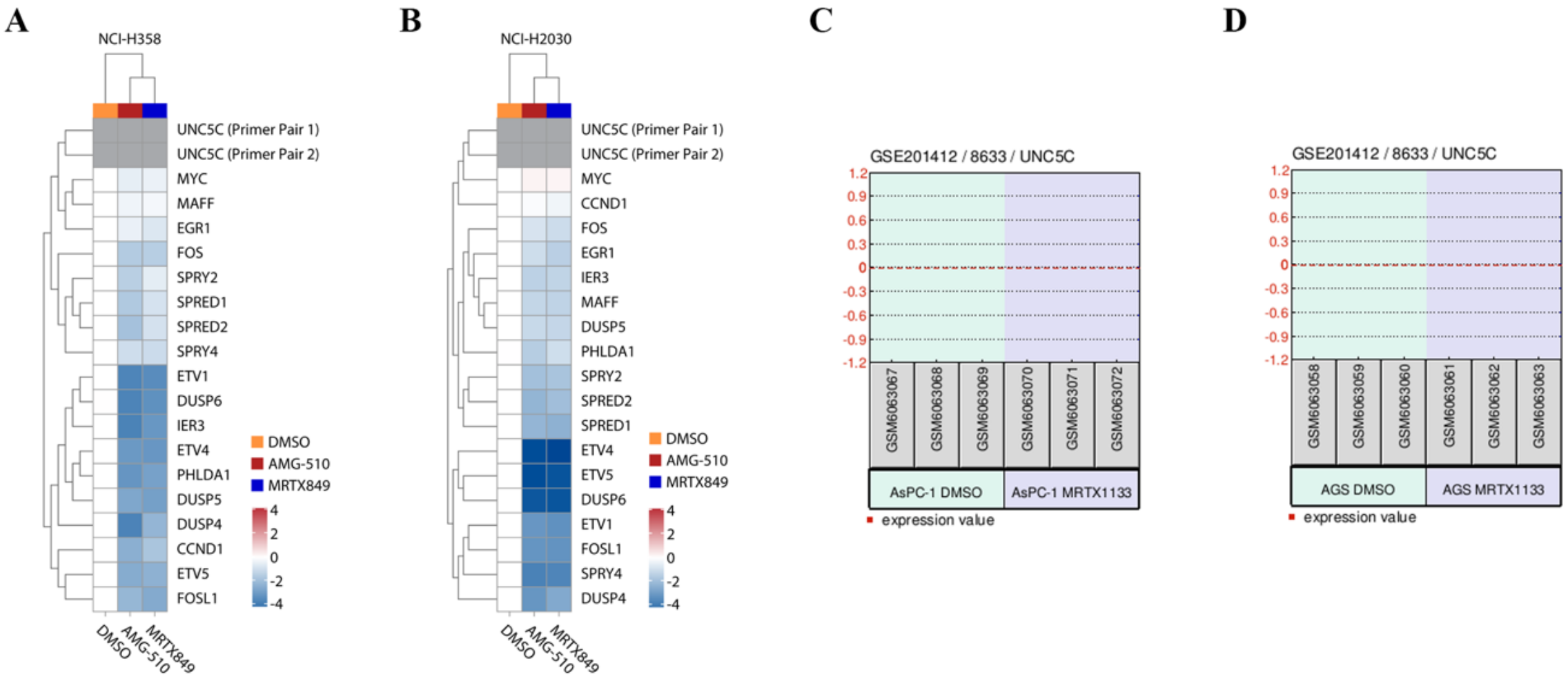

(A) Heatmap illustrating the relative expression of *UNC5C* and ERK-dependent genes in KRAS^{G12C}-mutant human NSCLC NCI-H358 cells after treatment by AMG-510 (1 μM) or MRTX849 (100 nM) for 24 h, as indicated by quantitative real-time PCR. Grey: Undetectable. (B) Heatmap illustrating the relative expression of *UNC5C* and ERK-dependent genes in KRAS^{G12C}-mutant human NSCLC NCI-H2030 cells after treatment by AMG-510 (1 μM) or MRTX849 (100 nM) for 24 h, as indicated by quantitative real-time PCR. Grey: Undetectable. (C) The expression values of UNC5C in KRAS^{G12D}-mutant human metastatic pancreatic adenocarcinoma AsPC-1 cells after exposure to KRAS^{G12D}-specific inhibitor MRTX1133 (100 nM) for 24 h from the public RNA-seq dataset (Jill Hallin et al. GSE201412)(17). (D) The expression values of UNC5C in KRAS^{G12D}-mutant human gastric cancer AGS cells after exposure to KRAS^{G12D}-specific inhibitor MRTX1133 (100 nM) for 24 h from the public RNA-seq dataset (Jill Hallin et al. GSE201412)(17).

Figure S4. Knockdown of oncogenic KRAS induced upregulation of UNC5C in KRAS-mutant NCI-H23 cells, related to Figure 2.

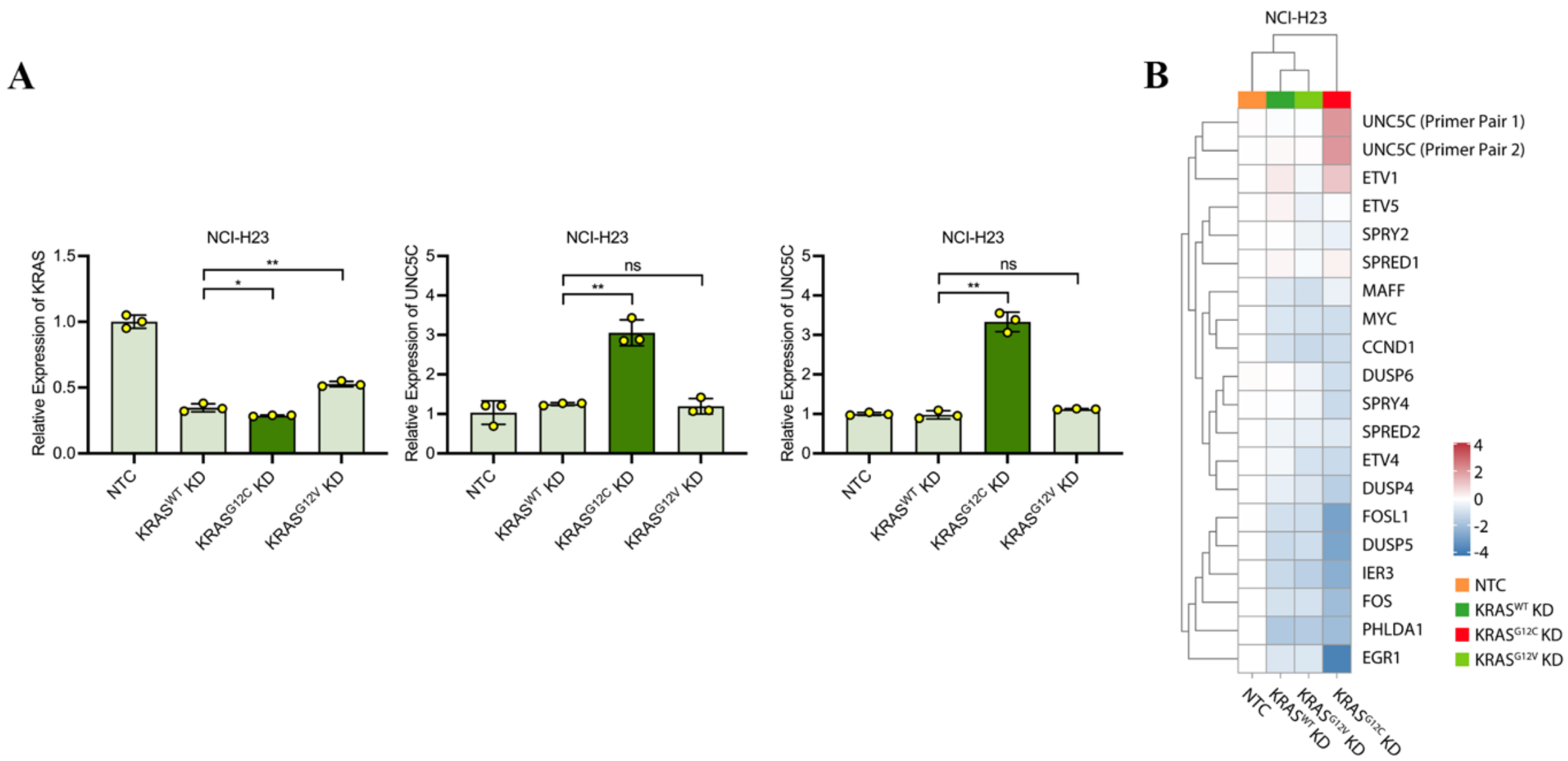

(A) Bar graph showing the relative expression of *KRAS* and *UNC5C* at mRNA levels after $KRAS^{G12C}$ knockdown in $KRAS^{G12C}$-mutant NCI-H23 cells. (B) Heatmap depicting the expression of *UNC5C* and ERK-dependent genes at mRNA levels in response to $KRAS^{G12C}$ knockdown in $KRAS^{G12C}$-mutant NCI-H23 cells. $*P<0.05$; $**P<0.01$.

Figure S5. The protein stability of UNC5C upon KRAS inhibitor treatment or oncogenic KRAS overexpression, related to Figure 2.

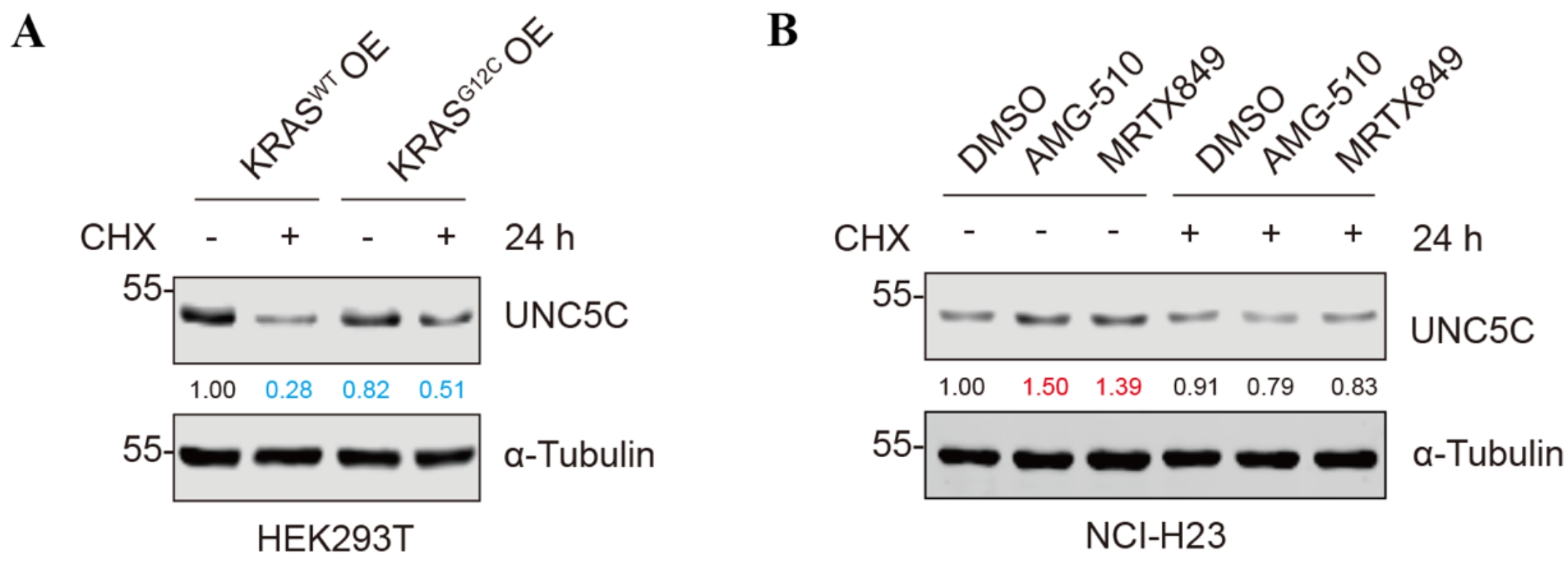

(A) CHX chase assay of UNC5C protein stability upon $KRAS^{WT}$ or oncogenic $KRAS^{G12C}$ overexpression in HEK293T cells. The cells were transiently transfected with $KRAS^{WT}$ or $KRAS^{G12C}$ plasmid. At 48 h after transfection, the cells were cultured in the absence or presence of CHX (5 μg/mL) as indicated for an additional 24 h. (B) CHX chase assay of UNC5C protein stability upon KRAS inhibitor AMG-510 or MRTX849 treatment in NCI-H23 cells. NCI-H23 cells were pretreated with or without KRAS inhibitor (either AMG-510 or MRTX849) for 24 h. After medium replacement, the cells were repeatedly treated with or without KRAS inhibitor in the absence or presence of CHX (5 μg/mL) as indicated for another 24 h. OE: overexpression.

Figure S6. Inhibitors of RAF/MEK/ERK pathway significantly and constantly induced upregulation of *UNC5C* expression in NCI-H23 cells, related to Figure 3.

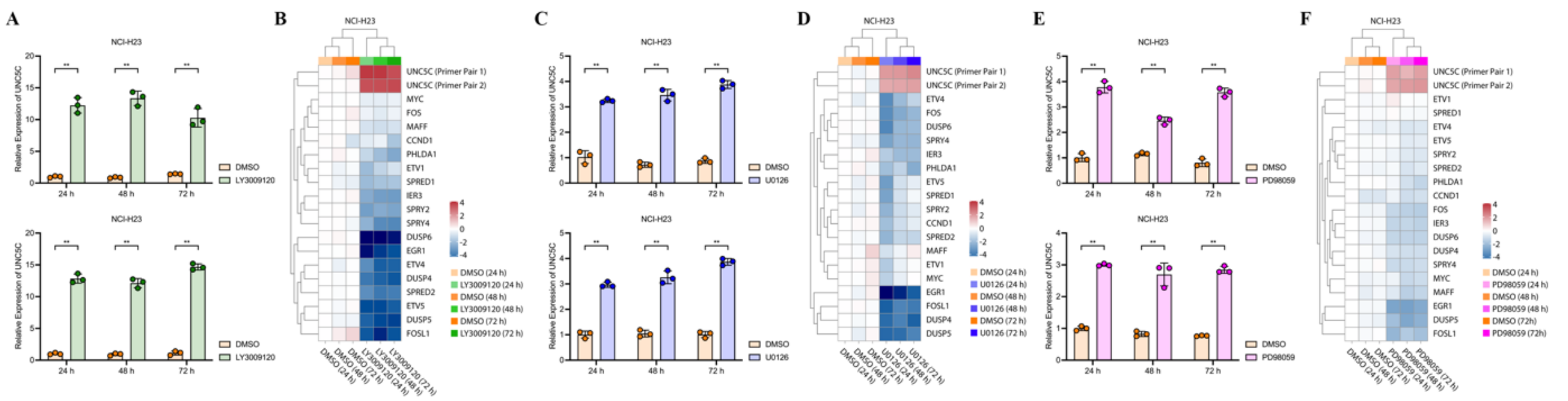

(A) Bar graph showing the relative expression of *UNC5C* in NCI-H23 cells after exposure to a pan-RAF inhibitor LY3009120 (10 μM) for the indicated time periods, as indicated by quantitative real-time PCR. (B) Heatmap illustrating the expression of *UNC5C* and ERK-dependent genes in NCI-H23 cells after exposure to a pan-RAF inhibitor LY3009120 (10 μM) for the indicated time periods, as indicated by quantitative real-time PCR. (C) Bar graph showing the relative expression of *UNC5C* in NCI-H23 cells after exposure to a MEK/ERK inhibitor U0126 (10 μM) for the indicated time periods, as indicated by quantitative real-time PCR. (D) Heatmap illustrating the expression of *UNC5C* and ERK-dependent genes in NCI-H23 cells after exposure to a MEK/ERK inhibitor U0126 (10 μM) for the indicated time periods, as indicated by quantitative real-time PCR. (E) Bar graph showing the relative expression of *UNC5C* in NCI-H23 cells after exposure to a MEK/ERK inhibitor PD98059 (20 μM) for the indicated time periods, as indicated by quantitative real-time PCR. (F) Heatmap illustrating the expression of *UNC5C* and ERK-dependent genes in NCI-H23 cells after exposure to a MEK/ERK inhibitor PD98059 (20 μM) for the indicated time periods, as indicated by quantitative real-time PCR. ***P*<0.01.

Figure S7. UNC5C was co-expressed with mesenchymal signature genes in tumor tissues of NSCLC patients, related to Figure 5.

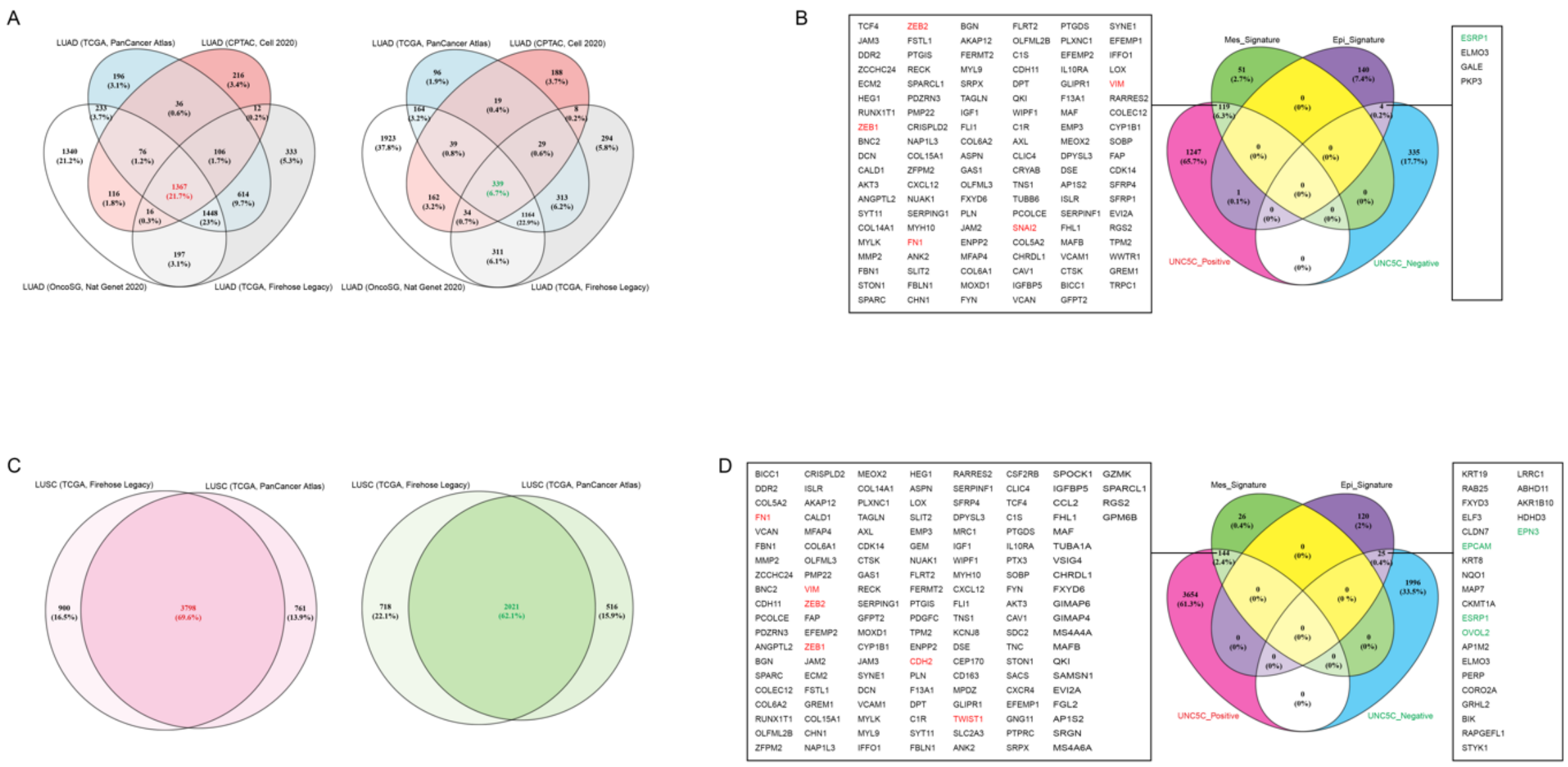

(A) Venn diagram depicting the overlap of genes positively (left panel, in red) or negatively (right panel, in green) correlated with UNC5C expression at mRNA levels across LUAD (OncoSG, Nat Genet 2020), LUAD (TCGA, Firehose Legacy), LUAD (TCGA, PanCancer Atlas), and LUAD (CPTAC, Cell 2020) datasets from the cBioportal (https://www.cbioportal.org/). (B) Venn diagram illustrating the overlap between gene positively or negatively correlated with UNC5C expression across LUAD datasets and epithelial or mesenchymal genes derived from generic EMT signature for tumors (18). (C) Venn diagram depicting the overlap of positively (left panel, in red) or negatively (right panel, in green) correlated with UNC5C expression at mRNA levels across LUSC (TCGA, Firehose Legacy) and LUSC (TCGA, PanCancer Atlas) datasets from the cBioportal (https://www.cbioportal.org/). (D) Venn diagram illustrating the overlap between gene positively or negatively correlated with UNC5C expression across LUSC datasets and epithelial or mesenchymal genes derived from generic EMT signature for tumors (18).

Reference

1. Shin, S. K., Nagasaka, T., Jung, B. H., Matsubara, N., Kim, W. H., Carethers, J. M. *et al.* (2007) Epigenetic and genetic alterations in Netrin-1 receptors UNC5C and DCC in human colon cancer *Gastroenterology* **133**, 1849-1857
2. Zhang, X., Cui, P., Ding, B., Guo, Y., Han, K., Li, J. *et al.* (2018) Netrin-1 elicits metastatic potential of non-small cell lung carcinoma cell by enhancing cell invasion, migration and vasculogenic mimicry via EMT induction *Cancer Gene Ther* **25**, 18-26
3. Zeng, Z., Yu, J., Jiang, Z., and Zhao, N. (2022) Oleanolic Acid (OA) Targeting UNC5B Inhibits Proliferation and EMT of Ovarian Cancer Cell and Increases Chemotherapy Sensitivity of Niraparib *J Oncol* **2022**, 5887671
4. Misale, S., Fatherree, J. P., Cortez, E., Li, C., Bilton, S., Timonina, D. *et al.* (2019) KRAS G12C NSCLC Models Are Sensitive to Direct Targeting of KRAS in Combination with PI3K Inhibition *Clin Cancer Res* **25**, 796-807
5. Hong, S. K., Wu, P. K., Karkhanis, M., and Park, J. I. (2015) ERK1/2 can feedback-regulate cellular MEK1/2 levels *Cell Signal* **27**, 1939-1948
6. Montero, J., Gstalder, C., Kim, D. J., Sadowicz, D., Miles, W., Manos, M. *et al.* (2019) Destabilization of NOXA mRNA as a common resistance mechanism to targeted therapies *Nat Commun* **10**, 5157
7. Kagoya, Y., Nakatsugawa, M., Saso, K., Guo, T., Anczurowski, M., Wang, C. H. *et al.* (2018) DOT1L inhibition attenuates graft-versus-host disease by allogeneic T cells in adoptive immunotherapy models *Nat Commun* **9**, 1915
8. Hou, Y., Lin, B., Xu, T., Jiang, J., Luo, S., Chen, W. *et al.* (2024) The neurotransmitter calcitonin gene-related peptide shapes an immunosuppressive microenvironment in medullary thyroid cancer *Nat Commun* **15**, 5555
9. DeNicola, G. M., Karreth, F. A., Humpton, T. J., Gopinathan, A., Wei, C., Frese, K. *et al.* (2011) Oncogene-induced Nrf2 transcription promotes ROS detoxification and tumorigenesis *Nature* **475**, 106-109
10. Sunaga, N., Kaira, K., Imai, H., Shimizu, K., Nakano, T., Shames, D. S. *et al.* (2013) Oncogenic KRAS-induced epiregulin overexpression contributes to aggressive phenotype and is a promising therapeutic target in non-small-cell lung cancer *Oncogene* **32**, 4034-4042
11. Kaplan, F. M., Shao, Y., Mayberry, M. M., and Aplin, A. E. (2011) Hyperactivation of MEK-ERK1/2 signaling and resistance to apoptosis induced by the oncogenic B-RAF inhibitor, PLX4720, in mutant N-RAS melanoma cells *Oncogene* **30**, 366-371
12. Cheung, L. W., Yu, S., Zhang, D., Li, J., Ng, P. K., Panupinthu, N. *et al.* (2014) Naturally occurring neomorphic PIK3R1 mutations activate the MAPK pathway, dictating therapeutic response to MAPK pathway inhibitors *Cancer Cell* **26**, 479-494
13. Chen, Y., Gorelik, G. J., Strickland, F. M., and Richardson, B. C. (2010) Decreased ERK and JNK signaling contribute to gene overexpression in "senescent" CD4+CD28- T cells through epigenetic mechanisms *J Leukoc Biol* **87**, 137-145
14. Carcamo-Orive, I., Tejados, N., Delgado, J., Gaztelumendi, A., Otaegui, D., Lang, V. *et al.* (2008) ERK2 protein regulates the proliferation of human mesenchymal stem cells without affecting their mobilization and differentiation potential *Exp Cell Res* **314**, 1777-1788

15. Hong, S. K., Yoon, S., Moelling, C., Arthan, D., and Park, J. I. (2009) Noncatalytic function of ERK1/2 can promote Raf/MEK/ERK-mediated growth arrest signaling *J Biol Chem* **284**, 33006-33018
16. Comandante-Lou, N., Baumann, D. G., and Fallahi-Sichani, M. (2022) AP-1 transcription factor network explains diverse patterns of cellular plasticity in melanoma cells *Cell Rep* **40**, 111147
17. Hallin, J., Bowcut, V., Calinisan, A., Briere, D. M., Hargis, L., Engstrom, L. D. *et al.* (2022) Anti-tumor efficacy of a potent and selective non-covalent KRAS(G12D) inhibitor *Nat Med* **28**, 2171-2182
18. Tan, T. Z., Miow, Q. H., Miki, Y., Noda, T., Mori, S., Huang, R. Y. *et al.* (2014) Epithelial-mesenchymal transition spectrum quantification and its efficacy in deciphering survival and drug responses of cancer patients *EMBO Mol Med* **6**, 1279-1293
